# Identification of *Rickettsia* spp. in Ticks Removed from Tick-Bitten Humans in Northwestern Spain

**DOI:** 10.3390/insects15080571

**Published:** 2024-07-27

**Authors:** María Carmen Vieira Lista, María Belén Vicente Santiago, Julio David Soto-López, Joaquina María García-Martín, Rufino Álamo-Sanz, Moncef Belhassen-García, Antonio Muro

**Affiliations:** 1 Infectious and Tropical Diseases Research Group (e-INTRO), Biomedical Research Institute of Salamanca-Research Centre for Tropical Diseases (IBSAL-CIETUS), Faculty of Pharmacy, University of Salamanca, 37008 Salamanca, Spain; carmelilla@usal.es (M.C.V.L.); jdjuliosoto@usal.es (J.D.S.-L.); kina@usal.es (J.M.G.-M.); belhassen@usal.es (M.B.-G.); 2 Consejería de Sanidad Junta Castilla y León, 47007 Valladolid, Spain; rufino.alamo@jcyl.es; 3 Infectious Diseases Unit, Department of Internal Medicine, University Hospital of Salamanca, 37008 Salamanca, Spain

**Keywords:** Ixodidae, *Rickettsia* spp., tick bites, tick-borne disease, zoonoses

## Abstract

**Simple Summary:**

We conducted a tick surveillance study in northwestern Spain with the aim of identifying tick-associated *Rickettsia* species among ticks removed from humans and to track how tick populations and their associated pathogens have changed over the years. Five genera and eight species of ticks were collected, with *Ixodes ricinus* being the most frequently found. By comparing our data with previous reports, a clear geographic and seasonal expansion of tick populations and the associated *Rickettsia* species is observed, indicating that the spatiotemporal patterns of ticks and bacteria have changed over the years. Considering the medical relevance of tick-borne rickettsioses, evaluating the infection risk to humans in tick-infested areas, as well as detecting spreading species, is essential for establishing adequate prevention and control measures.

**Abstract:**

Tick-borne rickettsioses (TBRs) are distributed worldwide and are recognized as important emerging vector-borne zoonotic diseases in Europe. The aim of this study was to identify tick-associated *Rickettsia* among ticks removed from humans, and to track how tick populations and their associated pathogens have changed over the years. For this purpose, we conducted a tick surveillance study in northwestern Spain between 2018 and 2022. Ticks were morphologically identified and analyzed for the presence of rickettsial pathogens through the amplification of the citrate synthase (*gltA*) and the outer membrane protein A (*ompA*) genes. PCR products were sequenced and subjected to phylogenetic analyses. We collected 7397 ticks, with *Ixodes ricinus* being the species most frequently isolated. Based on the PCR results, *Rickettsia* DNA was detected in 1177 (15.91%) ticks, and 10 members of *Rickettsia* were identified: *R. aeschlimannii*, *R. conorii* subsp. *conorii*, *R. conorii* subsp. *raoultii*, *R. massiliae*, *R. monacensis*, *R. sibirica* subsp. *mongolitimonae*, *R. slovaca*, *R. helvetica*, *Candidatus* R. barbariae, and *Candidatus* R. rioja. Some of these *Rickettsia* have gone previously undetected in the study region. There is clear geographic and seasonal expansion not only of tick populations, but also of the associated *Rickettsia*. The comparison of our data with those obtained years ago provides a clear idea of how the spatiotemporal distributions of ticks and their associated Rickettsiae have changed over the years.

## 1. Introduction

Rickettsioses are worldwide zoonoses caused by intracellular Gram-negative bacteria of the genera *Rickettsia* and *Orientia* (order Rickettsiales, family Rickettsiaceae). These infectious diseases are transmitted by different arthropod vectors, such as lice, fleas, mites, and ticks, the latter being involved in the transmission of most pathogenic Rickettsiae to humans. Tick-borne rickettsioses (TBRs) are distributed worldwide and are recognized as important emerging vector-borne zoonotic diseases in Europe [1,2], with cases being exclusively due to members of *Rickettsia* [3,4].

The genus *Rickettsia* currently comprises more than 30 species of intracellular coccobacilli bacteria, validly published under the International Code of Nomenclature of Prokaryotes (ICNP), and almost 90 candidate species (https://lpsn.dsmz.de/genus/Rickettsia; accessed on 20 April 2024). Among them, half are human pathogens [5], and eight are transmitted by different species of ticks in Europe [6]. Both pathogenic and nonpathogenic *Rickettsia* species have been classified into five distinct well-supported clades: (i) two spotted fever groups (SFGI and SFGII, also known as transitional groups (TRGs)); (ii) the typhus group (TG); (iii) the basal bellii group (BG); (iv) the canadensis group (CG); and (v) the Tamurae/Ixodes Group (TIG) [7,8,9]. SFGs are the most numerous group, with more than 56 validated and candidate species [10], although this number will likely change over time as novel SFG *Rickettsia* species continue to be identified.

In Europe, most SFG Rickettsiae are transmitted by ticks of the family Ixodidae. Specifically, the genera *Dermacentor*, *Haemaphysalis*, *Hyalomma*, *Ixodes*, and *Rhipicephalus* are considered the principal sources of infections naturally transmitted by ticks [11]. According to the European Centre for Disease Prevention and Control (ECDC), most cases of rickettsiosis have been reported in Italy, Portugal, and Spain.

In the case of the Iberian Peninsula, eight tick-borne *Rickettsia* species of medical concern have been detected: (1) *Rickettsia conorii* subsp. *conorii*, the etiological agent of Mediterranean spotted fever (MSF), transmitted by *Rhipicephalus sanguineus*; (2) *Rickettsia slovaca*, *Rickettsia conorii* subsp. *raoultii*, and *Candidatus* Rickettsia rioja, associated with the so-called *Dermacentor* spp.-borne necrosis-erythema-lymphadenopathy (DEBONEL); (3) *Rickettsia aeschlimannii*, *Rickettsia helvetica*, *Rickettsia monacensis*, and *Rickettsia massiliae*, all causing Mediterranean spotted fever-like illness (MSF-like illness); and (4) *Rickettsia sibirica* subsp. *mongolitimonae*, which causes lymphangitis-associated rickettsiosis (LAR) and is transmitted by *Rhipicephalus bursa* and *Hyalomma marginatum* [2,11,12].

Focusing on Castilla y León (northwestern Spain), the second largest regions in Europe, with a wide variety of ecosystems and bioclimatic conditions, there is a recent study on human-biting tick species and their spatial and temporal distributions [13], but their associated rickettsial pathogens were not analyzed in this study. Indeed, as far as we are aware, there is only an exhaustive study on ticks removed from people in Castilla y León and their associated rickettsial pathogens by members of our group, but it was carried out in the period 1997–2002 [14]. Since then, no additional studies have been conducted. The observed changes in tick populations by Vieira Lista et al. [13] make it essential to re-examine the current situation of pathogens transmitted by tick populations in this area.

The main purpose of this study was to fill this knowledge gap by identifying tick-associated *Rickettsia* species in ticks recently removed from humans in Castilla y León and comparing them with those found in the same area in the past. By doing so, we will be able to examine the spatiotemporal changes in the presence of ticks and their pathogens. Ultimately, this will allow us to evaluate the risk of TBR in tick-infested areas, as well as to detect spreading species and emerging diseases, which is essential for revealing its epidemiology and establishing adequate prevention and control measures.

## 2. Materials and Methods

### 2.1. Tick Collection and Identification

**Figure 1:**
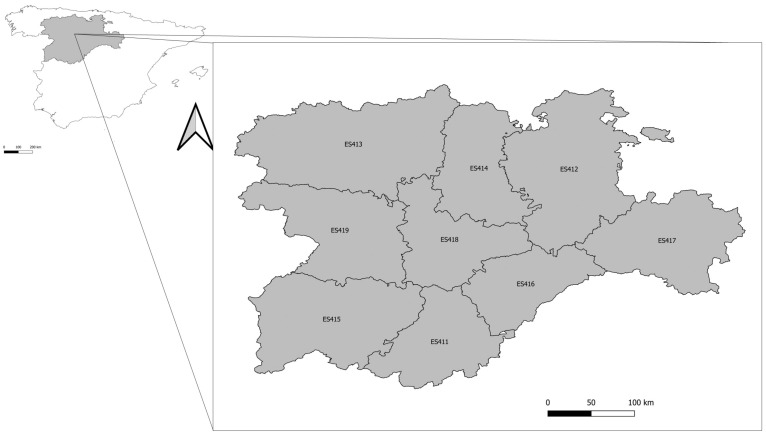
Map of the area of study (Castilla y León, Spain). Different provinces within Castilla y León are named according to NUTS regions: Ávila (ES411), Burgos (ES412), León (ES413), Palencia (ES414), Salamanca (ES415), Segovia (ES416), Soria (ES417), Valladolid (ES418), and Zamora (ES419).

Ticks were collected over a 5-year period (2018–2022) from people who went to Primary Health Care Centres and Hospital Emergency Services in Castilla y León (northwestern Spain) (Figure 1), for their removal through a program for the prevention and control of tick-borne anthropozoonoses.

Ticks were sent to the Laboratory of Infectious and Tropical Diseases (e-INTRO), Faculty of Pharmacy at the University of Salamanca (Spain), for identification and pathogen detection through molecular analyses. At their arrival, each tick was morphologically identified under a binocular lens according to life stage and sex using taxonomic keys and reference works [15,16,17]. Each specimen was identified to the species level, except for those ticks corresponding to *Rh. sanguineus sensu lato*, which were identified only to the group level due to the taxonomic issues of this species [18].

For each tick received, other data, such as sex and degree of feeding, as well as the epidemiological characteristics of the patients (age, sex, geographic location at the time of the bite(s), occupation, and the anatomical location of the tick bite), were collected.

All ticks examined are kept at −20 °C in the tick collection of the Laboratory of Infectious and Tropical Diseases of the University of Salamanca (Salamanca, Spain).

### 2.2. DNA Extraction, Amplification, Purification and Sequencing

Genomic DNA was extracted from ticks using a NucleoSpin^®^ Blood Kit (Macherey-Nagel GmbH & Co. KG, Düren, Germany) according to the manufacturer’s instructions. DNA was individually extracted from adult ticks. Only when several immature ticks were removed from the same patient, a DNA pool was made (specifically, six pools were prepared with 15 immature ticks removed from six people). DNA extractions were stored at −20 °C until use.

To detect the presence of rickettsial genetic material in the DNA samples obtained from the ticks analyzed, we used the citrate synthase gene (*gltA*) and the outer membrane protein A gene (*ompA*), which are present in all members of the SFG but not in close relatives [19]. Both genes were targeted by conventional PCR, including negative and positive controls in each assay, using previously reported primers [20,21] and protocols (Tables S1 and S2). Amplification reactions were performed in a Biometra^®^ T-personal Thermocycler (Whatman Biometra, Göttingen, Germany).

PCR products were visualized on a 1.5% agarose gel under a UVP Biodoc-It^®^ 2 imaging system (Analytik Jena, Jena, Germany). All positive PCR products were purified using a Nucleospin^®^ Gel and PCR Clean-up kit (Macherey-Nagel GmbH & Co. KG, Düren, Germany) and quantified with a NanoDrop ND-1000 Spectrophotometer (Nanodrop Technologies, Wilmington, NC, USA) with ND-1000 V3.8.1 software. Once purified, the amplicons were Sanger sequenced in one direction at the Sequencing Service of the University of Salamanca, Nucleus (Salamanca, Spain), with the same primer sets used for amplification.

### 2.3. Sequence Edition, Alignment and Phylogenetic Analyses

Sequence editing, which involved primer region trimming and consensus assembly, was performed in Geneious v7.1.9. (Biomatters, Ltd, Auckland, New Zealand; www.geneious.com). Edited sequences were subjected to BLAST searches against the NCBI GenBank database to verify that they did not correspond to ticks, and the sequences were assigned a putative identity based on the BLAST results (Table S3). Sequences that were too short relative to the global length of the alignment were not considered for subsequent phylogenetic analyses. All 581 new sequences used for phylogenetic purposes were submitted to GenBank under accession numbers PP552066–PP552646 (Table S4).

To place the samples analyzed in a phylogenetic context, 215 GenBank sequences obtained from species distributed among different groups within *Rickettsia* were downloaded and used for phylogenetic analyses (Table S5). Note that, aiming to cover the entire diversity of the genus, a greater number of GenBank sequences were initially included in exploratory analyses. However, long-branch attraction artefacts were identified (for example, for the sequences of *R. bellii* and *R. felis*), so they were not included in the final datasets. The sequences ultimately selected corresponded to 120 isolates for which *ompA* and *gltA* gene data were available, including 40 isolates designated “type strain”, which were used to delineate species within the genus *Rickettsia*, and 16 undetermined or uncultured *Rickettsia* species.

Homologous GenBank and newly obtained sequences were automatically aligned using MAFFT with the E-INS-i algorithm and default settings, as implemented in Geneious. Low-quality ends were trimmed before the alignment errors were manually adjusted. Both alignments used in this study are available as Supporting Information (Alignments S1 and S2).

For each gene, two common phylogenetic approaches, i.e., maximum likelihood (ML) and Bayesian inference (BI), were used to determine the phylogenetic position of the isolates obtained within the genus *Rickettsia*. We used different phylogenetic tools available in CIPRES (CyberInfrastructure for Phylogenetic Research; www.phylo.org). Specifically, single-gene ML trees were inferred using IQ-TREE v. 2.1.2 [22]. The best-fit model of nucleotide substitution and the optimal partitioning scheme for each gene (both were divided into three partitions corresponding to each codon position) were selected by the integrated version of ModelFinder [23]. The complete bootstrap option with 1000 nonparametric bootstrap replicates was used to determine bootstrap support (BS) for each node.

The BI individual analyses were performed using the Metropolis-coupled Markov chain Monte Carlo (MCMCMC) method, as implemented in MrBayes v. 3.2.7 [24]. For each gene dataset, the best-fit substitution model was estimated by sampling across the substitution model space with the reversible-jump Markov chain Monte Carlo (MCMC) method [25], allowing gamma distributed rate heterogeneity across sites and a proportion of invariant sites. The partitioning scheme selected by ModelFinder was used, unlinking model parameters across different partitions. Four independent runs of 20 million generations, each with six chains, were used, sampling trees every 1000 generations, with the first 25% being discarded as burn-in. The posterior probabilities (PPs) were calculated from the remaining probabilities. Bayesian analyses were automatically stopped when the average standard deviation of the split frequencies was smaller than 0.01. In addition, the convergence of the runs was checked in Tracer v. 1.7.2 [26] by checking that the effective sampling size (ESS) values for all parameters fell below 200. Single-gene trees were visually inspected in FigTree v. 1.4.4 [27] before being processed with Adobe Illustrator CS5 (Adobe Systems Inc., San Jose, CA, USA).

### 2.4. Statistical and Graphical Analyses

Fisher’s one-way repeated measures ANOVA (parametric) was used to estimate the difference in means of infection levels among tick species, life stages, months, and years. A post hoc test was performed with a Holm *p*-value correction due to multiple comparisons. A significant difference was assumed for values of *p* < 0.05. We estimated the effect size using Hedges’ G with a 95% confidence interval (CI). For the Bayesian interpretation, a Cauchy distribution r = 0.707 was considered a priory, the highest density interval (HDI) of 95% as credible intervals, and an interpretation of the logarithm of the Bayes factor according to the interpretation scales suggested by Jeffreys [28]. All the analyses were performed using the package ggstatsplot [29] in R [30].

To obtain a visual representation of the relationship between ticks and their associated *Rickettsia*, a heat map was made. First, we loaded the frequency of infected ticks of different species and converted these data into a matrix using R, and then the pheatmap function of the pheatmap package [31] was used to build the map.

## 3. Results

### 3.1. Tick Identification

A total of 7397 ticks removed from 7180 people were identified at the species level, with only *Rh. sanguineus* (*s.l.*) ticks being identified at the group level. The tick specimens identified included 140 larvae, 2227 nymphs, and 5030 adults belonging to five genera and eight species (Table 1). *Ixodes ricinus* was the most represented (427/3269, 44.19%).

In general, the highest number of tick bites was reported during spring and summer (April to July), with the peak occurring in May and June of most years. However, from 2021 onwards, the activity peak began earlier, in April, and remained very high until July.

### 3.2. Molecular Detection of Rickettsia

**Figure 2:**
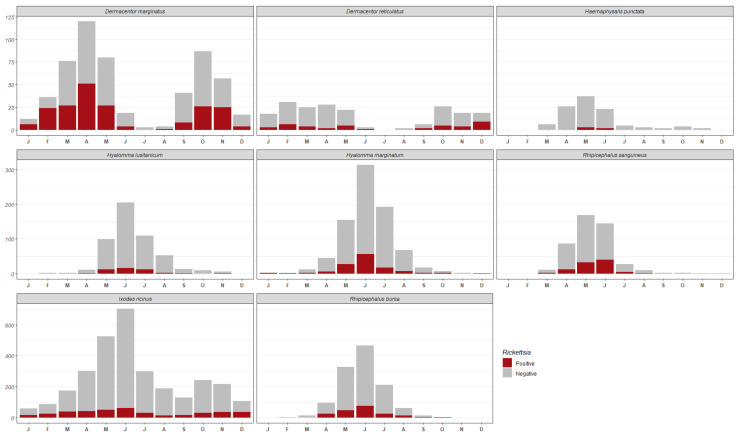
Number of *Rickettsia*-positive and *Rickettsia*-negative ticks per species versus months.

Rickettsial DNA was detected in 1177 of the 7397 ticks analyzed (Table 1). For these specimens, *gltA*, *ompA*, or both genes could be amplified, which represents an overall prevalence of 15.91%. Although *I. ricinus* was the species most frequently carrying *Rickettsia*, the highest percentage of infected specimens per species corresponded to the genera *Dermacentor* (*D. marginatus*, 35.88% and *D. reticulatus*, 20.19%), followed by *Rhipicephalus* (*Rh. sanguineus*, 19.5% and *Rh. bursa*, 16%) (Table 1). In terms of the percentage of ticks infected according to their life stage and sex, we found that 80% of the positive ticks were adults (54% females and 26% males), 1% were larvae, and 19% were nymphs. In terms of monthly differences, ticks infected by different *Rickettsia* species were detected throughout the year (Figure 2), with June being the month with the highest percentage of *Rickettsia*-positive ticks (24.06%), followed by May (19.73%) and April (12.8%). The interannual distribution of *Rickettsia*-carrying ticks was quite regular throughout the study period, showing a decreasing trend.

### 3.3. Sequences and BLAST (Identification of Rickettsia Species)

A total of 504 isolates out of the 1177 *Rickettsia*-positive samples were sequenced, resulting in 581 partial sequences (311 sequences corresponding to *ompA* and 270 to *gltA*, Table S4), which allowed the identification of 10 different taxa. Specifically, based on BLAST searches of the *ompA* gene sequences, all our tick-isolated *Rickettsia* sequences showed high similarity to GenBank sequences corresponding to nine different members of the spotted fever group I (SFGI): *R. aeschlimannii*, *R. conorii* subsp. *conorii*, *R. conorii* subsp. *raoultii*, *R. massiliae*, *R. monacensis*, *R. sibirica* subsp. *mongolitimonae*, *R. slovaca*, *Candidatus* R. barbariae, and Ca. R. rioja. Similarly, most isolates positive for the *gltA* gene matched with some *Rickettsia* species belonging to the SGFI, and 15 isolates matched with the species *R. helvetica*.

**Figure 3:**
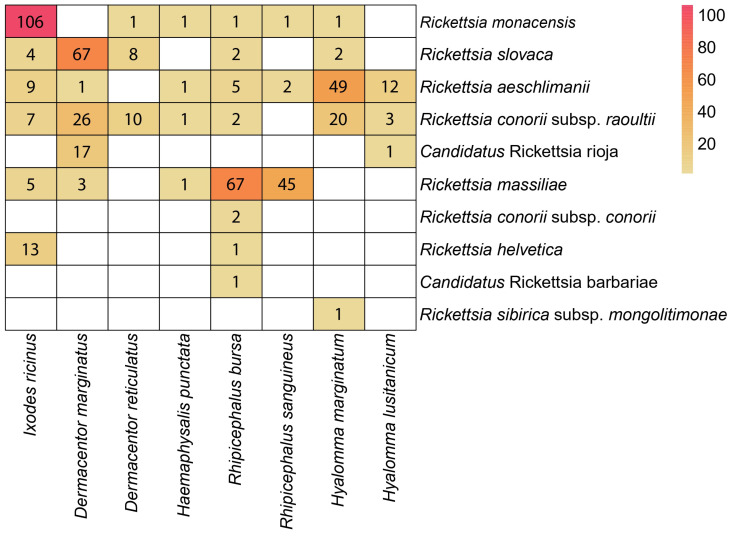
Heat map showing the number of *Rickettsia* isolates per tick species. Red represents the highest number of infected ticks and yellowish the smallest number.

According to BLAST analyses (Table S3), the most prevalent *Rickettsia* species was *R. massiliae* (24%, 121/504). It was detected mainly in ticks of the genus *Rhipicephalus* (*Rh. bursa*, 67/121 and *Rh. sanguineus*, 45/121) (Figure 3). The second most prevalent species was *R. monacensis*, with a prevalence of 22.22% (Table S3, 112/504), which was mainly detected in *I. ricinus* (106/111). These were followed by a group consisting of *R. slovaca*, *R. aeschlimanii*, and *R. conorii* subsp. *raoultii*, with prevalences of 16.43%, 15.63%, and 13.82%, respectively. *Rickettsia slovaca* and *R. conorii* subsp. *raoultii* were mainly detected in *D. marginatus*, and *Hy. marginatum* and *R. aeschmimanii* were detected in *Hy. marginatum*. In the case of Ca. R. rioja and *R. helvetica*, the prevalence rates were very similar (3.6% and 2.8%, respectively), showing in both cases a high specificity for the vector species: Ca. R. rioja was almost exclusively found in *D. marginatus* specimens, and *R. helvetica* was found in *I. ricinus*.

For the remaining members of *Rickettsia* found in this study (*R. conorii* subsp. *conorii*, Ca. R. barbariae, and *R. sibirica* subsp. *mongolitimonae*), their prevalence ranged from 0.2 to 0.4%, with each being found in only one tick species: *R. conorii* subsp. *conorii* and Ca. R. barbariae in *Rh. bursa*, and *R. sibirica* subsp. *mongolitimonae* in *Hy. marginatum*.

### 3.4. Phylogenetic Analyses

**Figure 4:**
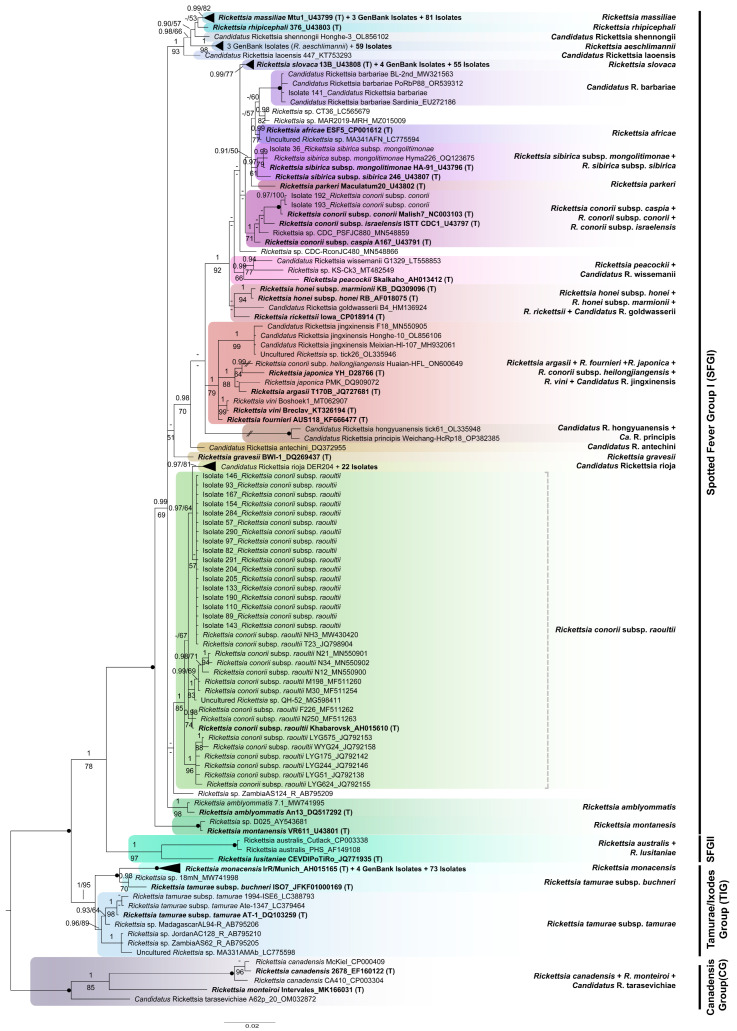
Bayesian phylogenetic tree of the rickettsial isolates obtained, based on 608 nucleotide positions of *ompA*, with members of the “Canadensis group” as outgroup. Bayesian posterior probabilities (PP) and maximum likelihood bootstrap support (BS) values are shown above and below each branch if PP > 0.90 and BS > 50%, respectively. Lower support values are represented by dashes. Solid dots indicate nodes fully supported (PP = 1, BS = 100%). For newly obtained sequences, the isolate number is followed by the species name. In the case of the GenBank sequences used as reference, the species name is followed by strain ID and accession number, with type strains appearing in bold and marked with a (T). For representation purposes, well-supported clades comprising isolates obtained here plus one type strain have been collapsed, thus appearing as solid black triangles. An interrupted branch (//) indicates its length has been reduced. Continuous and discontinuous vertical lines represent mono- and paraphyletic groups, respectively. The scale bar represents the average number of substitutions per site.

**Figure 5:**
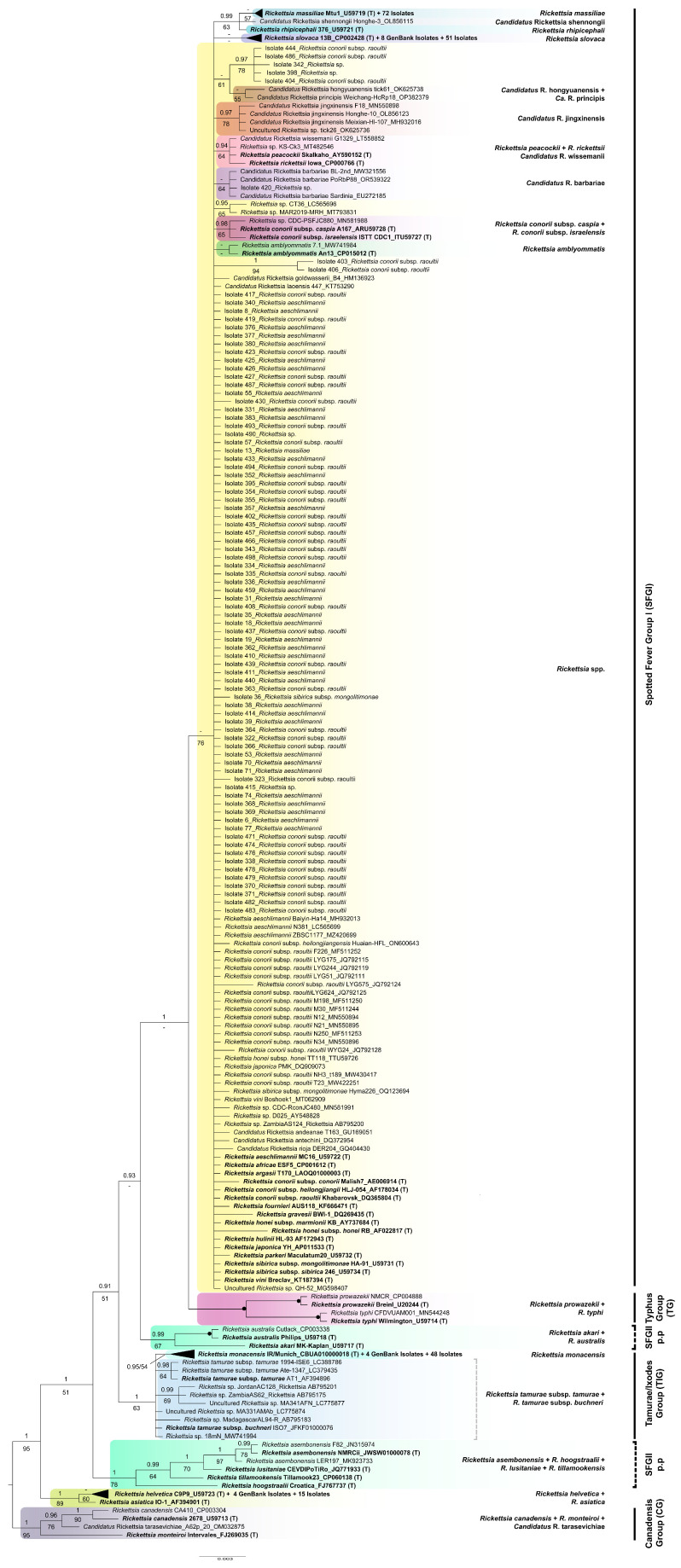
Bayesian phylogenetic tree of the rickettsial isolates obtained, based on 341 nucleotide positions of *gltA*, with members of the “Canadensis group” as outgroup. Bayesian posterior probabilities (PP) and maximum likelihood bootstrap support (BS) values are shown above and below each branch if PP > 0.90 and BS > 50%, respectively. Lower support values are represented by dashes. Solid dots indicate nodes fully supported (PP = 1, BS = 100%). For newly obtained sequences, the isolate number is followed by the species name. In the case of the GenBank sequences used as reference, the species name is followed by strain ID and accession number, with type strains appearing in bold and marked with a (T). For representation purposes, well-supported clades comprising isolates obtained here plus one type strain have been collapsed, thus appearing as solid black triangles. Continuous and discontinuous vertical lines represent mono- and paraphyletic groups, respectively. The scale bar represents the average number of substitutions per site.

After excluding primer binding regions and low-quality ends, the alignments used for phylogenetic purposes were 608 and 341 bp long (*ompA* and *gltA*, respectively). The optimal partitioning scheme and best-fit model of nucleotide substitution for each gene matrix used in our phylogenetic analyses are shown in Table S6. For both genes, both ML and BI analyses yielded similar topologies, so only the Bayesian tree showing PP and BS values is shown (Figure 4 and Figure 5). For better visualization, well-supported clades comprising a high number of isolates were collapsed (solid black triangles). The extended versions of these trees can be found in the Supplementary Material (Figures S1 and S2).

Individually, none of the two molecular regions analyzed confidently resolved all relationships among the newly obtained isolates and different *Rickettsia* species used as references, as indicated by the relatively low support values recovered for some internal branches in both single-gene trees (Figure 4, Figure 5 and Figures S1 and S2). This is especially true for the *gltA* tree (Figure 5 and Figure S2), with many isolates arising from a polytomy and containing sequences obtained from the type strains of several other species. However, in general, most of our isolates formed different well-defined species clusters in both the *ompA* and *gltA* trees. An interrupted branch (//) indicates its length has been reduced. Continuous and discontinuous vertical lines represent mono- and paraphyletic groups, respectively. The scale bar represents the average number of substitutions per site.

As shown in Figure 4, for illustrates, the genus *Rickettsia* is subdivided into four monophyletic groups: (1) CG (PP = 1, BS = 100%), (2) TIG (PP = 1, BS = 95%), (3) SGFII (PP = 1, BS = 97%), and (4) SGFI (PP = 1, BS = 100%). All 311 isolates for which *ompA* could be sequenced are distributed among different subgroups within SGFI, each comprising the sequence generated from the type strain of an accepted species of *Rickettsia*, which, thus, gives name to that subgroup. Specifically, our isolates were distributed among the following eight clades: (1) “*R. monacensis*” (PP = 1, BS = 100%; including 73 new isolates); (2) “*Ca.* R. rioja” (PP = 0.97, BS = 81%; 22 isolates); (3) “*Rickettsia conorii* subsp. *caspia* + *R. conorii* subsp. *conorii* + *R. conorii* subsp. *israelensis*” (PP = 1, BS = 71%; 2 isolates); (4) “*R. sibirica* subsp. *mongolitimonae*”, *R. sibirica* subsp. *sibirica*” (PP = 0.97, BS = 61%; 1 isolate); (5) “*Ca.* R. barbariae” (PP = 1, BS = 100%; 1 isolate); (6) “*Rickettsia slovaca*” (PP = 0.99, BS = 77%; 55 isolates); (7) “*R. aeschlimannii*” (PP = 1; BS = 98%; 59 isolates); and (8) “*R. massiliae*” (PP = 0.99, BS = 82%; 81 isolates). Additionally, 17 new isolates clustered within the paraphyletic group “*R. conorii* subsp. *raoultii*” (PP = 1; BS = 85%), from which the clade “*Ca.* R. rioja” was derived. A deep understanding of the phylogeny of the genus *Rickettsia* is beyond the scope of this study, so the relationships recovered among the species isolated will not be explained here.

The phylogenetic tree inferred from our *gltA* partial sequences (Figure 5 and Figure S2) is highly unresolved, as evidenced by the existence of a large poorly supported polytomy (PP = 0.74, BS = 76%), corresponding to the SFGI. The other main groups within the genus *Rickettsia* are either well or moderately supported, except for SFGII, which was recovered as polyphyletic. Specifically, the CG and the group formed by *R. helvetica* and *R. asiatica* were monophyletic with high support (PP = 1, BS = 95% and PP = 1, BS = 89%, respectively), the TIG was only moderately supported (PP = 1, BS = 63%), the typhus group (TG) was fully supported (PP = 1, BS = 100%), and the sister had low support for the polytomy (PP = 1, BS = 47%). Most of our isolates (207 out of 270) form part of the mentioned polytomy, with only some being included in the subgroups arising from it. In detail, one isolate (Isolate 420) clustered within the “*Ca.* R. barbariae” group, only moderately supported by ML (PP = 0.85, BS = 64%); three isolates identified as *Rickettsia conorii* subsp. *raoultii* by BLAST and two undetermined ones grouped together in a well-supported cluster (PP = 0.97, BS = 78%) that does not comprise any sequences from a type strain and, thus, is unnamed; 51 isolates form an unsupported group (PP = 0.55, BS = 15%) that includes the type strain of *R. slovaca*; and 72 additional ones clustered together with the type strain of *R. massiliae*, also forming an unsupported group (PP = 0.75, BS = 57%). The remaining isolates were distributed in different subgroups not arising from the polytomy as follows: 15 isolates clustered within the highly supported clade “*R. helvetica* + *R. asiatica*” (PP = 1, BS = 89%), and 48 isolates grouped together with the type strain of *R. monacensis* (PP = 0.95, BS = 54%).

### 3.5. Distribution of Rickettsia Species within the Sampling Area

**Figure 6:**
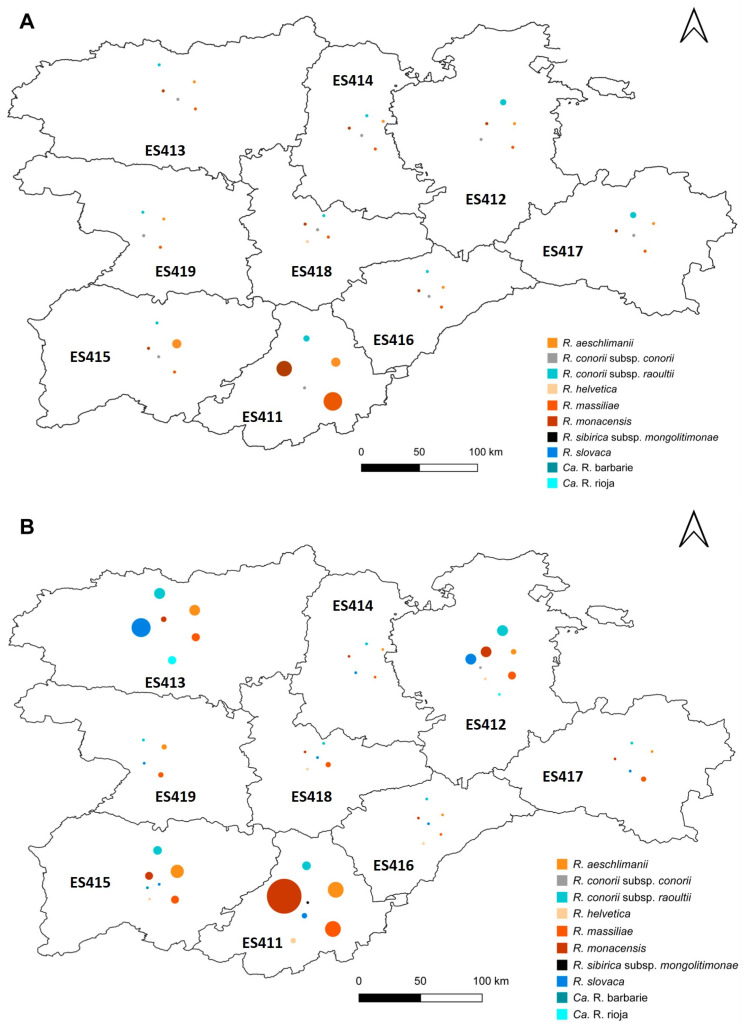
Distribution of *Rickettsia* species within the sampling area (Castilla y León, northwestern Spain), in two different periods. Each species is represented by a colored circle with size proportional to the number of isolates found. (**A**) Sampling period between 1997 and 2002 (previous study). (**B**) Sampling between 2018 and 2022 (this study).

The geographical distribution of some *Rickettsia* species is clearly delimited within Castilla y León (Figure 6). This is the case for Ca. R. rioja, since 100% of the ticks infected by this bacterium were found in the northwestern region. Additionally, in the northern region, we found 66.7% of all ticks that carry *R. slovaca* in Castilla y León.

Most *R. aeschlimanii* (60%), *R. helvetica* (71.39%), *R. massiliae* (47.9%), and *R. monacensis* (65.5%) isolates were recovered from ticks found in the southern part of the sampling area. In the case of the ticks infected by *R. conorii* subsp. *raoultii*, however, they were more evenly distributed throughout the study area. In turn, most ticks infected by *R. conorii* subsp. *conorii* were found in the north, and those infected by Ca. R. barbariae and *R. sibirica* subsp. *mongolitimonae* were found in the south, although these infections were very infrequent.

### 3.6. Statistical Analyses

**Figure 7:**
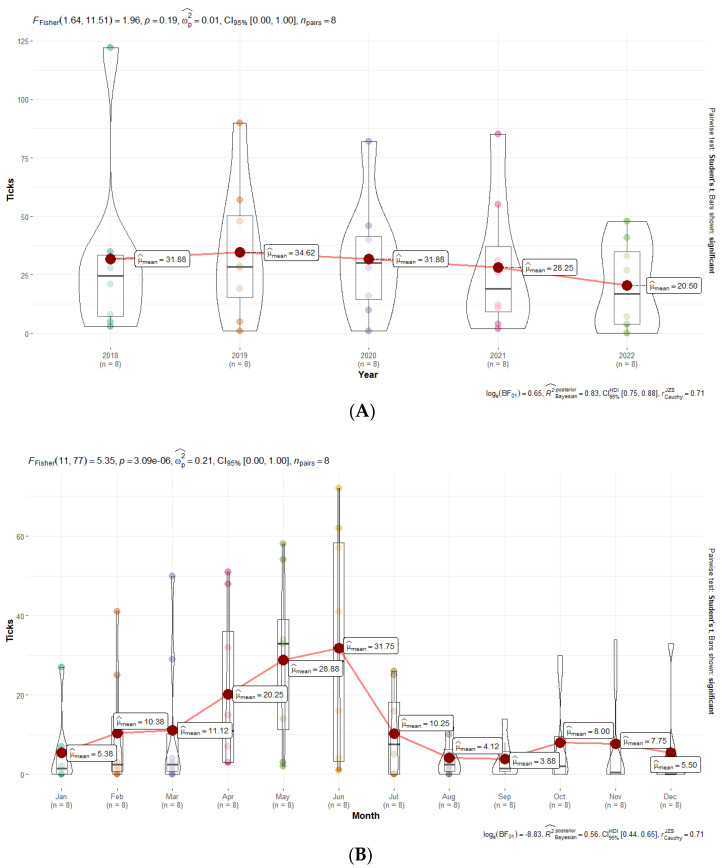
Differences in the number of infected ticks per year (**A**) and month (**B**).

Fisher’s one-way repeated measures ANOVA test revealed that, considering only the infected ticks, there were not statistically significant differences among different years (Figure 7A).

Although we originally observed statistically significant differences among months, after post hoc pairwise *t*-tests with the Holm *p*-value correction, no significant differences existed (Figure 7B).

**Figure 8:**
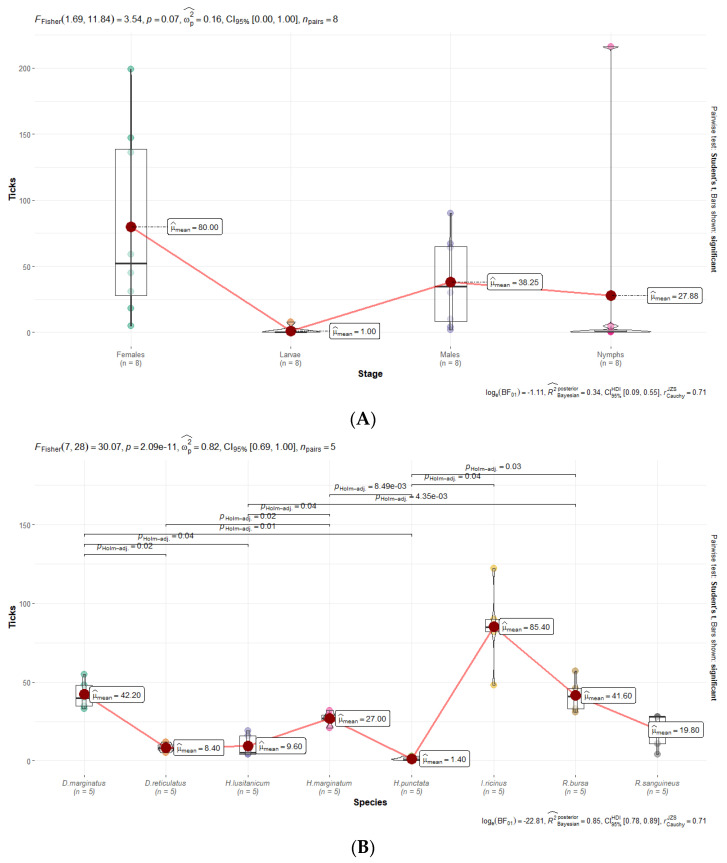
Differences in the number of infected ticks per stages (**A**) and species (**B**).

There were no statistically significant differences among different stages (Figure 8A), although they were observed among tick species (Figure 8B). The effect size (ωp = 0.82) was large, as per Field’s conventions [32]. The Bayes Factor for the same analysis revealed that the data were more probably under the alternative hypothesis, as compared to the null hypothesis (BF01-22.81). This can be considered very strong evidence in favor of the alternative hypothesis [28]. This global effect was carried out by post hoc pairwise *t*-tests, which revealed that there are significant differences among different species (Figure 8).

## 4. Discussion

Ticks and tick-borne diseases (TBDs), especially tick-borne rickettsioses (TBR), are a growing problem for human health and have been reported in different countries and regions, including neighboring countries to Spain, i.e., France and Portugal [33,34], among many others. Moreover, the incidence of emerging TBD, including rickettsiosis, is likely to increase in the near future [35,36]. For these reasons, studies addressing the surveillance of ticks and emerging and re-emerging TBDs, such as rickettsial diseases, are essential not only for identifying areas and populations at risk but also for implementing appropriate prevention and control measures. These prevention measures include using appropriate clothes and repellent against ticks when visiting risk areas, self-examination after outdoor activities, the removal of any ticks as soon as possible, and the deparasitation of domestic and farm animals.

The present study provides relevant information on the diversity and seasonal distribution of human-biting ticks in Castilla y León (northwest Spain) over a five-year span, as well as relevant molecular data on the tick-associated *Rickettsia* that they could transmit to humans in this region. Compared to what was observed by Fernández-Soto [14], the most evident difference is the changing distribution pattern of two species, i.e., *R. slovaca* and *R. monacensis*, within the study area. We have seen here that *R. slovaca* is now restricted to the north of Castilla y León, especially to the northwest region, while years ago, it was found in virtually the whole study area. However, in the case of *R. monacensis*, we have seen the opposite trend: previously, this species was almost restricted to a single locality in the south, while it is now found across Castilla y León, although it is still more prevalent in southern areas. It is also important to note that *R. monacensis* is the species that increased the most in number and distribution area.

We analyzed both the *ompA* and *gltA* benchmark genes and found that our isolates belong to different members of the SFGI and to the species *R. helvetica*, which is currently unassigned to a defined group within the genus.

Specifically, based on the information provided by *ompA* (Figure 4 and Figure S1), most of our isolates clustered within the SFGI and corresponded to Ca. *R.* rioja, Ca. *R.* barbarie, *R. aeschlimannii*, *R. conorii* subsp. *raoultii*, and *R. massiliae*. *R. monacensis*, and *R. slovaca*. Considering the phylogeny inferred from the *gltA* gene (Figure 5 and Figure S2), which is not resolved as evidenced by the polytomy of indeterminate relatedness, the existence of different independent well-supported clades adds some information to that provided by *ompA*: all 15 isolates identified as *R. helvetica* by BLAST form a group sister to *R. asiatica* with high support, as previously found using phylogenomic data [37]. Moreover, 48 *gltA* isolates belong to *R. monacensis*.

Focusing on the ticks biting people in the study area, *Ixodes ricinus* was clearly the predominant species (it was the one most frequently removed from people), followed by *Rh. bursa*, which coincides with the results obtained in other studies carried out by our group [13]. However, we detected an increase in the percentage of three tick species: *Hy. marginatum* (11.81% vs. 9.08%), *Hy. lusitanicum* (11.81 vs. 9.08%), and *Rh. bursa* (17.56% vs. 12.15%). This upwards trend was already noted during the last years of our previous study and has been maintained over time. For the remaining tick species, there were no significant changes in terms of abundance.

Notably, 15% of the ticks tested were positive for rickettsial DNA, a much greater percentage than that observed in a previous study (approximately 7%) carried out by our group in the same area years ago [14], but very similar to those more recently reported in some locations of Italy (15–17%) [38,39] and Poland [40]. This percentage is, however, much lower than those reported in other European countries, such as France [34], the Netherlands [41], Serbia [42], and Turkey [43], ranging from 22 to 44%. In contrast, in Sweden [44] and Romania [45], the prevalence rates are less than 9%.

We found that *Dermacentor marginatus* was the species with the highest prevalence of rickettsial infection (35.88%), which is consistent with what was observed in the same area by Fernández-Soto [14] but also in other regions of Spain [46,47], Italy [38], and France [34].

Seven members of the genus *Rickettsia*, i.e., *Rickettsia aeschlimannii*, *R. conorii* subsp. *conorii*, *R. conorii* subsp. *raoultii*, *R. massiliae*, *R. monacensis*, *R. helvetica* and *R. slovaca*, have been found to be associated with different species of ticks in this study but also in that previously conducted by Fernández-Soto [14] in the same area, which clearly indicates that all these bacteria were present in Castilla y León since at least 20 years ago. Nevertheless, we also found several *Rickettsia* species that were nonexistent, or at least not identified, in the mentioned study. This is the case for *Candidatus* R. rioja, *Candidatus* R. barbariae, and *R. sibirica* subsp. *mongolitimonae*. However, it is important to note that these species have also been found in regions close to the sampling area. Specifically, *Candidatus* R. rioja, an SFG *Rickettsia* species involved in DEBONEL/TIBOLA cases, has been detected in patients bitten by ticks in La Rioja, a region to the northeast of Castilla y León [48]. Similarly, *R. sibirica* subsp. *mongolitimonae* has been found in *Rh. bursa* removed from cattle and *Rh. pusillus* from rabbits in central Spain [49]. Most importantly, this species has been reported to cause human infections [33,50]. In regions more distant from the sampling area, *Candidatus* R. barbariae, originally named strain PoTiRb169, has also been detected in *Rh. bursa* ticks collected in Sardinia [51]. These findings, along with ours, confirm the broad distribution of these agents in southern Europe but also raise the possibility that some cases of rickettsiosis diagnosed as MSF, mostly caused by *R. conorii* subsp. *conorii*, could actually be due to other members of *Rickettsia*.

By comparing the *Rickettsia* species currently present in the study area with those previously reported, it can be seen that some of them have increased in number to the detriment of others that have decreased. In both periods (1997–2002 and 2018–2022), the most prevalent *Rickettsia* species in Castilla y León was *R. massiliae*, but curiously, there were differences in the tick vector species. In the study carried out between 1997 and 2002, all the ticks carrying *R. massiliae* belonged to the *Rh. sanguineus* complex, while, in our study, they belonged not only to the *Rh. sanguineus* complex but also to the species *Rh. bursa*. We detected more ticks infected by this *Rickettsia* species in the southern region of the community, which was expected considering that its main vectors are mainly found in this area; however, compared to what was reported in a previous study, the greatest increase in *Rickettsia* occurred in the northern provinces. This could be explained by the fact that both *Rh. bursa* and *Rh. sanguineus* are beginning to establish themselves in the northern provinces of the community [13].

According to our results, currently, the second most prevalent *Rickettsia* species is *R. monacensis*, which is somewhat different from what was reported in the study by Fernández-Soto [14], in which *R. slovaca* and *R. aeschlimanii* were more abundant. *Rickettsia monacensis*, mainly detected in *I. ricinus*, is the species that increased the most in terms of number and distribution range with respect to the mentioned study. Although it is still more abundant in the south, we have seen an increase in the north (northeast), which coincides with the increase in *I. ricinus* in this area.

The prevalence of ticks infected by *R. slovaca* and *R. aeschlimanii* has decreased compared with that found in a previous study, while the number of ticks infected by *R. conorii* subsp. *raoultii* has increased over time (Table S7). The explanation for this increase is that *R. conorii* subsp. *raoultii* shares vectors with the other two mentioned *Rickettsia* species. According to previous results [14], *R. conorii* subsp. *raoultii* was mostly transmitted by ticks of the genus *Dermacentor*, while our data indicate that it is now transmitted by ticks belonging to *Dermacentor*, but also to the genus *Hyalomma* in similar proportions, as depicted in Figure 3.

It is very important to highlight the role of *Hy. marginatum* as a vector of this *Rickettsia* species since, in a previous study, it was mainly transmitted by the genus *Dermacentor*. For the other two *Rickettsia* species, ticks of the genera *Dermacentor* (mainly *D. marginatus*) and *Hyalomma* (*Hy. marginatum*) were the main vectors of *R. slovaca* and *R. aeschlimanii*, respectively.

When studying the distribution of these *Rickettsia* species, we observed that *R. slovaca* continues to be predominant in the north, although it has moved to the west, as well as its main vector, *D. marginatus* [13], and that *R. aeschlimanii* has a very similar distribution to that seen years ago, with a higher prevalence in the south and an absence in the center of the community. *Rickettsia helvetica* was less prevalent in the current study than in that by Fernández-Soto [14], although in both studies, it was found almost exclusively in *I. ricinus* and mostly in the southern region.

*Ca. R.* rioja was detected for the first time in ticks attached to humans in Castilla y León in 2019. It should be noted that this uncultivated species, which is phylogenetically very close to *R. conorii* subsp. *raoultii*, can only be distinguished from the latter by analysis of *ompA* because most authors include it under *R. conorii* subsp. *raoultii* [5]. However, since 2019, the incidence of *Candidatus* R. rioja has increased until 2022, when the highest number of specimens of this genus of *Rickettsia* was detected. This species is confined to the northern zone, although, as we have seen for *R. slovaca*, it is moving westwards. This geographic shift seems logical since both *Candidatus* R. rioja and *R. slovaca* share the same vector. 

The species with the greatest number of ticks infected by *Rickettsia* species was *Rh. bursa* (eight species), followed by *I. ricinus* (six), *D. marginatus*, and *Hy. marginatum* (five). These results are quite different from those observed by Fernández-Soto [14], who identified *I. ricinus* as the main vector species associated with six different *Rickettsia* species.

The results of this study show important aspects related to human-biting ticks and their associated pathogenic *Rickettsia*. However, this is only a partial vision of the human–animal–environment interface. Considering that the health of humans, animals, and ecosystems are interconnected, it is urgently needed to obtain a whole picture of the actual situation, including the most medically/veterinary important pathogens transmitted by ticks. Future directions include multidisciplinary collaborations and cross-sectoral studies for a better understanding of this multifaceted problem and to be able to tackle the potential emergence of tick-related zoonotic diseases.

## 5. Conclusions

This long-term study represents a step forward to a better understanding of the *Rickettsia* associated with ticks in northwestern Spain. Despite the fact that it was impossible to molecularly characterized all ticks positive for *Rickettsia*, our comprehensive morphological study of the ticks and the identification of a considerable number of bacterial isolates, at molecular level, allow us to have a clear vision of the epidemiological situation in the region.

Our results show the presence of a variety of pathogenic *Rickettsia* in ticks removed from humans in Castilla y León, including several species that have gone undetected to date. We have observed a wider geographical and seasonal expansion of tick populations than previously acknowledged in this area, as well as an increase in the prevalence of ticks carrying *Rickettsia*, with a consequent risk to human health.

When comparing our results with those obtained in previous studies conducted in the same area, evident changes in the distribution patterns of different rickettsial species were observed. *Ixodes ricinus* and *Rh. bursa* are the predominant tick species removed from humans. Ten different members of the genus *Rickettsia* (nine SFGI and *R. helvetica*) were detected in the ticks collected in this study, with *R. massiliae* being the most prevalent species and *R. monacensis* being the species with the greatest increase in number and distribution area.

## Figures and Tables

**Table 1:** Data on the ticks analyzed, including tick species, number of specimens collected for each species, and number of specimens carrying *Rickettsia* species.

Tick Species	No. Specimens Analysed (%)	No. Positive for *Rickettsia*	Percentage of Infected Specimens per Species
*Dermacentor marginatus* (Sulzer, 1776)	588 (7.95%)	211	35.88%
*Dermacentor reticulatus* (Fabricius, 1794)	208 (2.81%)	42	20.19%
*Haemaphysalis punctata* (Canestrini and Fanzago, 1877)	121 (1.64%)	7	5.79%
*Hyalomma lusitanicum* (Koch, 1844)	531 (7.18%)	48	9.04%
*Hyalomma marginatum* (Koch, 1844)	874 (11.82%)	135	15.45%
*Ixodes ricinus* (Linnaeus, 1758)	3269 (44.19%)	427	13.06%
*Rhipicephalus bursa* (Canestrini and Fanzago, 1877)	1299 (17.56%)	208	16.01%
*Rhipicephalus sanguineus* (Latreille, 1806)	507 (6.85%)	99	19.53%
Total	7397 (100%)	1177	15.91%

## Data Availability

All data supporting the findings of this study are present within this article and online Supplementary Materials. The sequences generated are deposited in GenBank. Any additional data are available on request to the corresponding author.

## Supplementary Materials

The following supporting information can be downloaded at: https://www.mdpi.com/article/10.3390/insects15080571/s1, Figure S1: Bayesian phylogenetic tree of the rickettsial isolates obtained, based on 608 nucleotide positions of *ompA*; Figure S2: Bayesian phylogenetic tree of the rickettsial isolates obtained, based on 341 nucleotide positions of *gltA*; Table S1: Primers used for PCR amplification and size of the amplicons; Table S2: Cycling parameters used in this study; Table S3: Rickettsial isolates obtained from different tick species, and putative identity based on BLAST results of *ompA* and *gltA* partial sequences; Table S4: Rickettsial isolates newly obtained from different tick species in this study; Table S5: GenBank sequences generated from 120 strains, included in the phylogenetic analyses conducted in this study; Table S6: Best partition scheme and substitution model(s) for both datasets used in phylogenetic analyses, chosen according to BIC; Table S7: Comparison of the prevalence of three *Rickettsia* species in different periods in Castilla y León. Alignment S1: alignment of *ompA* gene sequences; Alignment S2: alignment of *gltA* gene sequences. References in Supplementary Materials: [7,14,20,21,37].

## References

[1] Oteo J.A., Portillo A. (2012). Tick-borne rickettsioses in Europe. Ticks Tick Borne Dis..

[2] Remesar S., Fernández P.D., Venzal J.M., Pérez-Creo A., Prieto A., Estrada-Peña A., López C.M., Panadero R., Fernández G., Díez-Baños P. (2019). Tick species diversity and population dynamics of *Ixodes ricinus* in Galicia (north-western Spain). Ticks Tick Borne Dis..

[3] Portillo A., Santibáñez S., García-Álvarez L., Palomar A.M., Oteo J.A. (2015). Rickettsioses in Europe. Microbes Infect..

[4] Guccione C., Colomba C., Tolomeo M., Trizzino M., Iaria C., Cascio A. (2021). Rickettsiales in Italy. Pathogens.

[5] Remesar S., Cano-Terriza D., Morrondo P., Jiménez-Ruiz S., López C.M., Jiménez-Martín D., Díaz P., Paniagua J., García-Bocanegra I. (2023). Molecular detection of *Rickettsia* spp. in wild ungulates and their ticks in Mediterranean areas of southwestern Spain. Zoonoses Public Health.

[6] Maitre A., Wu-Chuang A., Mateos-Hernández L., Foucault-Simonin A., Moutailler S., Paoli J.C., Falchi A., Díaz-Sánchez A.A., Banović P., Obregón D. (2022). *Rickettsia helvetica* infection is associated with microbiome modulation in *Ixodes ricinus* collected from humans in Serbia. Sci. Rep..

[7] El Karkouri K., Ghigo E., Raoult D., Fournier P.-E. (2022). Genomic evolution and adaptation of arthropod-associated *Rickettsia*. Sci. Rep..

[8] Fournier P.-E., Raoult D. (2009). Current knowledge on phylogeny and taxonomy of *Rickettsia* spp. Ann. N. Y. Acad. Sci..

[9] Merhej V., Raoult D. (2011). Rickettsial evolution in the light of comparative genomics. Biol. Rev. Camb. Philos. Soc..

[10] Igolkina Y., Nikitin A., Verzhutskaya Y., Gordeyko N., Tikunov A., Epikhina T., Tikunova N., Rar V. (2023). Multilocus genetic analysis indicates taxonomic status of “*Candidatus* Rickettsia mendelii” as a separate basal group. Ticks Tick Borne Dis..

[11] Moerbeck L., Domingos A., Antunes S. (2022). Tick-borne rickettsioses in the Iberian Peninsula. Pathogens.

[12] Romaní Vidal A., Fernández-Martínez B., Herrador Z., León Gómez I., Gómez Barroso D. (2020). Spatial and temporal trends of Mediterranean spotted fever in Spain, 2005–2015. Ticks Tick Borne Dis.

[13] Vieira Lista M.C., Belhassen-García M., Vicente Santiago M.B., Sánchez-Montejo J., Pedroza Pérez C., Monsalve Arteaga L.C., Herrador Z., del Álamo-Sanz R., Benito A., Soto López J.D. (2022). Identification and distribution of human-biting ticks in Northwestern Spain. Insects.

[14] Fernández-Soto P. (2003). Garrapatas Que Parasitan a Las Personas en Castilla y León, Determinación Por Serología de su Parasitismo y Detección Molecular de Los Patógenos Que Albergan.

[15] Gil-Collado J., Guillen J., Zapatero L. (1979). Claves para la identificacion de los Ixodoidea espanoles (adultos). Rev. Ibérica Parasitol..

[16] Guglielmone A.A., Robbins R.G., Apanaskevich D.A., Petney T.N., Estrada-Peña A., Horak I.G. (2014). The Hard Ticks of the World (Acari: Ixodida: Ixodidae).

[17] Estrada-Peña A., Martinez J.M., Sanchez Acedo C., Quilez J., Del Cacho E. (2004). Phenology of the tick, *Ixodes ricinus*, in its southern distribution range (central Spain). Med. Vet. Entomol..

[18] Dantas-Torres F., Latrofa M.S., Annoscia G., Giannelli A., Parisi A., Otranto D. (2013). Morphological and genetic diversity of *Rhipicephalus sanguineus sensu lato* from the New and Old Worlds. Parasit. Vectors.

[19] Fournier P.-E., Roux V., Raoult D. (1998). Phylogenetic analysis of spotted fever group rickettsiae by study of the outer surface protein rOmpA. Int. J. Syst. Evol. Microbiol..

[20] Roux V., Rydkγna E., Eremeeva M., Raoult D. (1997). Citrate synthase gene comparison, a new tool for phylogenetic analysis, and its application for the Rickettsiae. Int. J. Syst. Evol. Microbiol..

[21] Regnery R.L., Spruill C.L., Plikaytis B.D. (1991). Genotypic identification of rickettsiae and estimation of intraspecies sequence divergence for portions of two rickettsial genes. J. Bacteriol..

[22] Nguyen L.-T., Schmidt H.A., von Haeseler A., Minh B.Q. (2015). IQ-TREE: A fast and effective stochastic algorithm for estimating maximum-likelihood phylogenies. MBE.

[23] Kalyaanamoorthy S., Minh B.Q., Wong T.K.F., von Haeseler A., Jermiin L.S. (2017). ModelFinder: Fast model selection for accurate phylogenetic estimates. Nat. Methods.

[24] Ronquist F., Teslenko M., van der Mark P., Ayres D.L., Darling A., Höhna S., Larget B., Liu L., Suchard M.A., Huelsenbeck J.P. (2012). MrBayes 3.2: Efficient Bayesian phylogenetic inference and model choice across a large model space. Syst. Biol..

[25] Huelsenbeck J.P., Larget B., Alfaro M.E. (2004). Bayesian phylogenetic model selection using reversible jump Markov chain Monte Carlo. MBE.

[26] Rambaut A., Drummond A.J., Xie D., Baele G., Suchard M.A. (2018). Posterior summarization in Bayesian phylogenetics using Tracer 1.7. Syst. Biol..

[27] Rambaut A. (2018). FigTree v 1.4.4. https://github.com/rambaut/figtree/releases/tag/v1.4.4.

[28] Jeffreys H. (1961). The theory of probability. Oxford Classic Texts. The Physical Sciences.

[29] Patil I. (2021). Visualizations with statistical details: The’ggstatsplot’approach. J. Open Source Softw..

[30] R Core Team (2024). R: A Language and Environment for Statistical Computing.

[31] Kolde R., Kolde M.R. (2015). Package ‘pheatmap’. R Package.

[32] Field A. (2013). Discovering Statistics Using IBM SPSS Statistics.

[33] de Sousa R., Barata C., Vitorino L., Santos-Silva M., Carrapato C., Torgal J., Walker D., Bacellar F. (2006). *Rickettsia sibirica* isolation from a patient and detection in ticks, Portugal. Emerg. Infect. Dis..

[34] Aubry C., Socolovschi C., Raoult D., Parola P. (2016). Bacterial agents in 248 ticks removed from people from 2002 to 2013. Ticks Tick Borne Dis..

[35] de la Fuente J., Estrada-Peña A., Rafael M., Almazán C., Bermúdez S., Abdelbaset A.E., Kasaija P.D., Kabi F., Akande F.A., Ajagbe D.O. (2023). Perception of ticks and tick-borne diseases worldwide. Pathogens.

[36] Piotrowski M., Rymaszewska A. (2020). Expansion of tick-borne rickettsioses in the world. Microorganisms.

[37] Verhoeve V.I., Fauntleroy T.D., Risteen R.G., Driscoll T.P., Gillespie J.J. (2022). Cryptic genes for interbacterial antagonism distinguish *Rickettsia* species infecting blacklegged ticks from other *Rickettsia* pathogens. Front. Cell Infect. Microbiol..

[38] Otranto D., Dantas-Torres F., Giannelli A., Latrofa M.S., Cascio A., Cazzin S., Ravagnan S., Montarsi F., Zanzani S.A., Manfredi M.T. (2014). Ticks infesting humans in Italy and associated pathogens. Parasit. Vectors.

[39] Audino T., Pautasso A., Bellavia V., Carta V., Ferrari A., Verna F., Grattarola C., Iulini B., Pintore M.D., Bardelli M. (2021). Ticks infesting humans and associated pathogens: A cross-sectional study in a 3-year period (2017–2019) in northwest Italy. Parasit. Vectors.

[40] Kubiak K., Dmitryjuk M., Dziekońska-Rynko J., Siejwa P., Dzika E. (2022). The risk of exposure to ticks and tick-borne pathogens in a spa town in northern Poland. Pathogens.

[41] Jahfari S., Hofhuis A., Fonville M., van der Giessen J., van Pelt W., Sprong H. (2016). Molecular detection of tick-borne pathogens in humans with tick bites and erythema migrans, in the Netherlands. PLoS Negl. Trop. Dis..

[42] Banović P., Díaz-Sánchez A.A., Simin V., Foucault-Simonin A., Galon C., Wu-Chuang A., Mijatović D., Obregón D., Moutailler S., Cabezas-Cruz A. (2022). Clinical aspects and detection of emerging rickettsial pathogens: A “one health” approach study in Serbia, 2020. Front. Microbiol..

[43] Gargili A., Palomar A.M., Midilli K., Portillo A., Kar S., Oteo J.A. (2012). *Rickettsia* species in ticks removed from humans in Istanbul, Turkey. Vector Borne Zoonotic Dis..

[44] Lindblom A., Wallménius K., Sjöwall J., Fryland L., Wilhelmsson P., Lindgren P.E., Forsberg P., Nilsson K. (2016). Prevalence of *Rickettsia* spp. in ticks and serological and clinical outcomes in tick-bitten individuals in Sweden and on the Åland Islands. PLoS ONE.

[45] Andersson M.O., Marga G., Banu T., Dobler G., Chitimia-Dobler L. (2018). Tick-borne pathogens in tick species infesting humans in Sibiu County, central Romania. Parasitol. Res..

[46] Merino F.J., Nebreda T., Serrano J.L., Fernández-Soto P., Encinas A., Pérez-Sánchez R. (2005). Tick species and tick-borne infections identified in population from a rural area of Spain. Epidemiol. Infect..

[47] Oteo J., Portillo A., Santibáñez S., Pérez-Martínez L., Blanco J., Jiménez S., Ibarra V., Pérez-Palacios A., Sanz M. (2006). Prevalence of spotted fever group *Rickettsia* species detected in ticks in La Rioja, Spain. Ann. N. Y. Acad. Sci..

[48] Ibarra V., Oteo J., Portillo A., Santibanez S., Blanco J., Metola L., Eiros J., Pérez-Martínez L., Sanz M. (2006). Rickettsia slovaca infection: DEBONEL/TIBOLA. Ann. N. Y. Acad. Sci..

[49] Toledo A., Olmeda A.S., Escudero R., Jado I., Valcárcel F., Casado-Nistal M.A., Rodríguez-Vargas M., Gil H., Anda P. (2009). Tick-borne zoonotic bacteria in ticks collected from central Spain. Am. J. Trop. Med. Hyg..

[50] Aguirrebengoa K., Portillo A., Santibáñez S., Marín J.J., Montejo M., Oteo J.A. (2008). Human *Rickettsia sibirica mongolitimonae* infection, Spain. Emerg. Infect. Dis..

[51] Mura A., Masala G., Tola S., Satta G., Fois F., Piras P., Rolain J.-M., Raoult D., Parola P. (2008). First direct detection of rickettsial pathogens and a new rickettsia, ‘*Candidatus* Rickettsia barbariae’, in ticks from Sardinia, Italy. Clin. Microbiol. Infect..

