# Supplementary material for: Identification of Rickettsia spp. in Ticks Removed from Tick-Bitten Humans in Northwestern Spain
Source: Insects. 2024 Jul 27;15(8):571. doi: 10.3390/insects15080571 (PMC11354666; doi:10.3390/insects15080571)
Supplement: Supplementary file 1 [file insects-15-00571-s001.zip › insects-3057412 -Supplementary_Material_proofread.pdf]

SUPPLEMENTARY FIGURES

Figure S1. Bayesian phylogenetic tree of the Rickettsial isolates obtained, based on 608 nucleotide positions of *ompA*, with members of the "Canadensis group" as outgroup. Bayesian posterior probabilities (PP) and maximum likelihood bootstrap support (BS) values are shown above and below each branch if PP > 0.90 and BS > 50%, respectively. Lower support values are represented by dashes. Solid dots indicate nodes fully supported (PP = 1, BS = 100%). For newly obtained sequences, the isolate number is followed by the species name. In the case of the GenBank sequences used as reference, the species name is followed by strain ID, and accession number, with type strains appearing in bold and marked with a (T). An interrupted branch (//) indicates its length has been reduced. Continuous and discontinuous vertical lines represent mono- and paraphyletic groups, respectively. The scale bar represents the average number of substitutions per site.

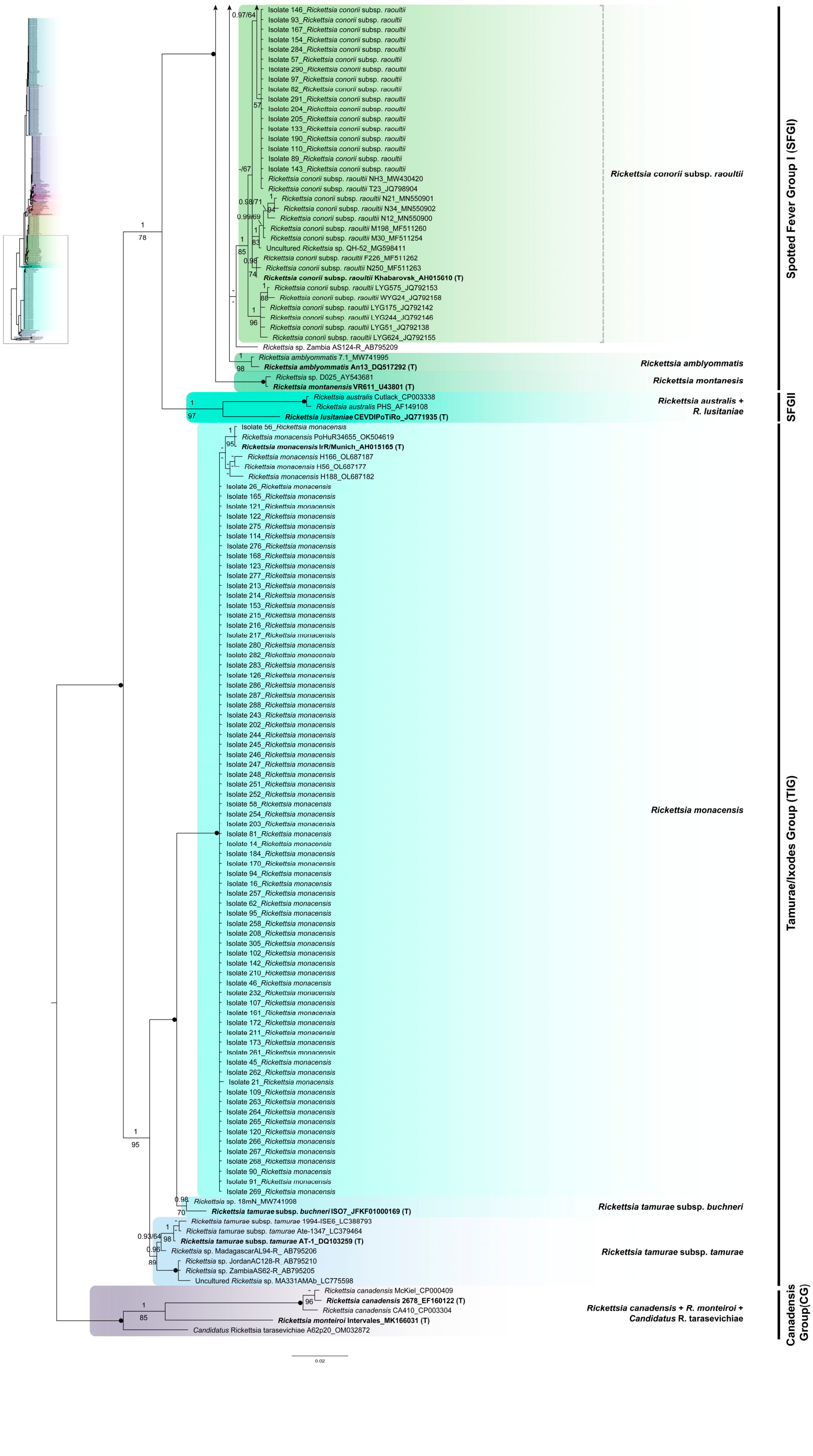

Figure S1. (Continued).

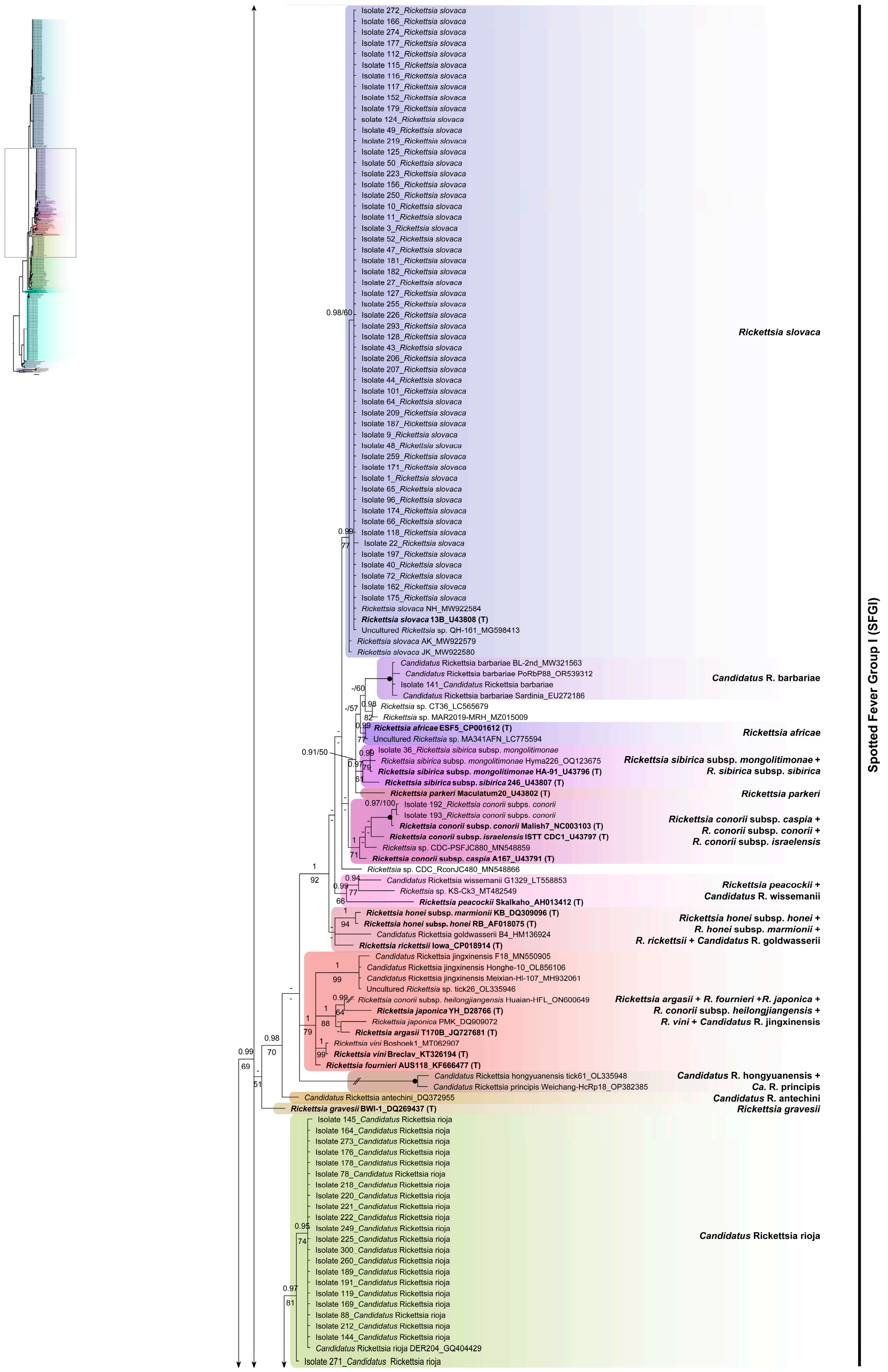

Figure S1. (Continued).

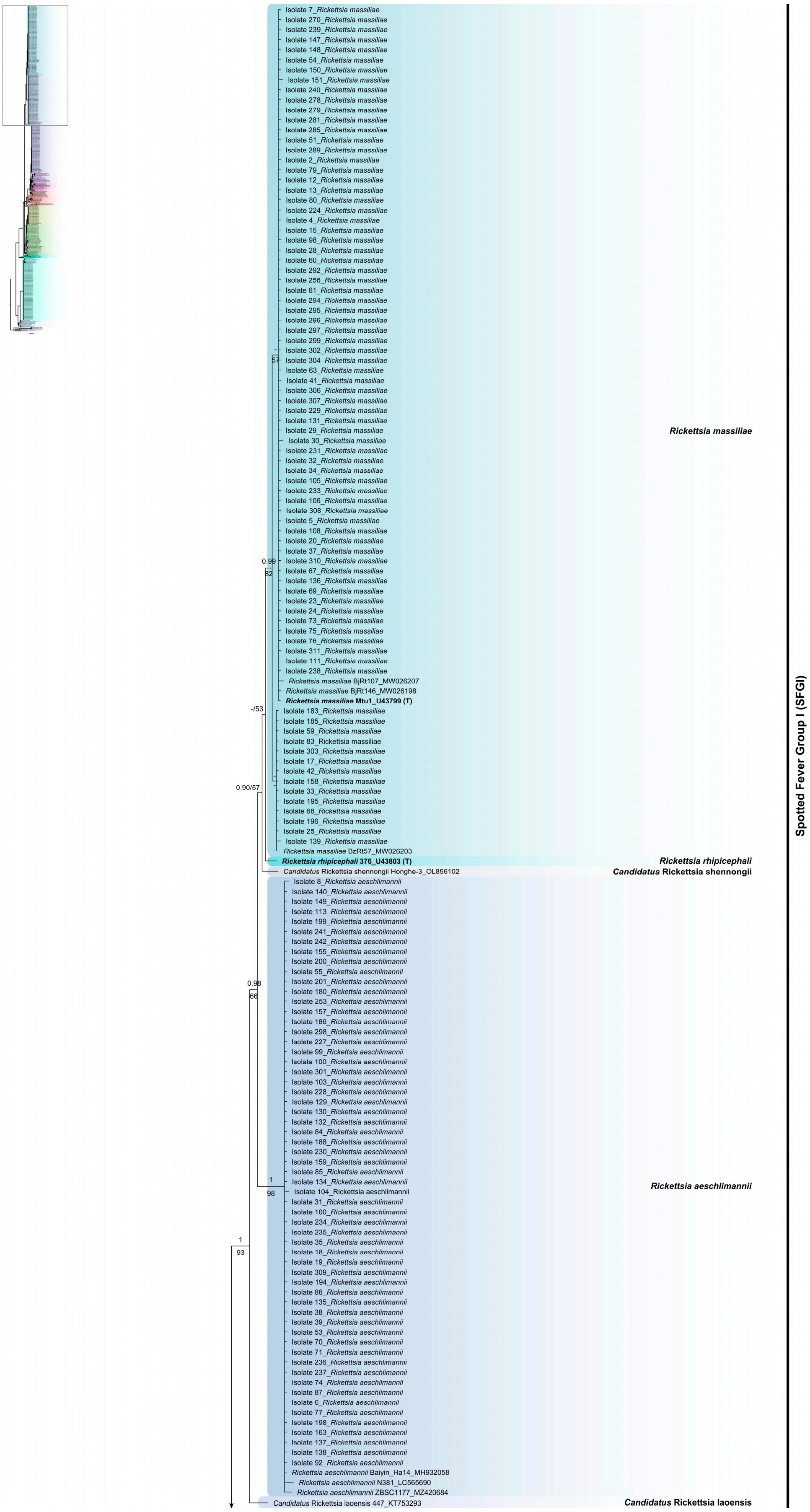

Figure S2. Bayesian phylogenetic tree of the Rickettsial isolates obtained, based on 341 nucleotide positions of *gltA*, with members of the "Canadensis group" as outgroup. Bayesian posterior probabilities (PP) and maximum likelihood bootstrap support (BS) values are shown above and below each branch if PP > 0.90 and BS > 50%, respectively. Lower support values are represented by dashes. Solid dots indicate nodes fully supported (PP = 1, BS = 100%). For newly obtained sequences, the isolate number is followed by the species name. In the case of the GenBank sequences used as reference, the species name is followed by strain ID, and accession number, with type strains appearing in bold and marked with a (T). An interrupted branch (//) indicates its length has been reduced. Continuous and discontinuous vertical lines represent mono- and paraphyletic groups, respectively. The scale bar represents the average number of substitutions per site.

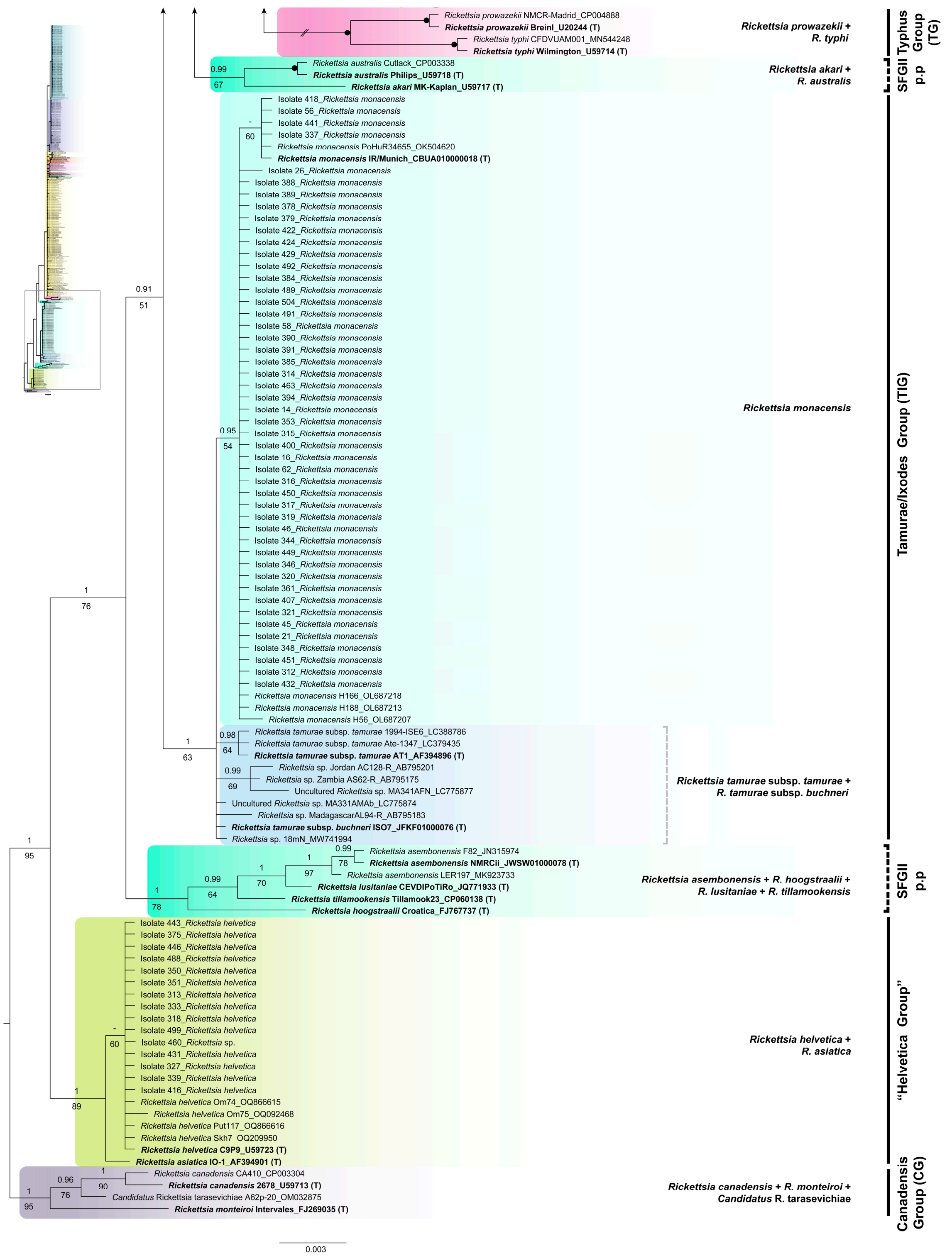

Figure S2. (Continued).

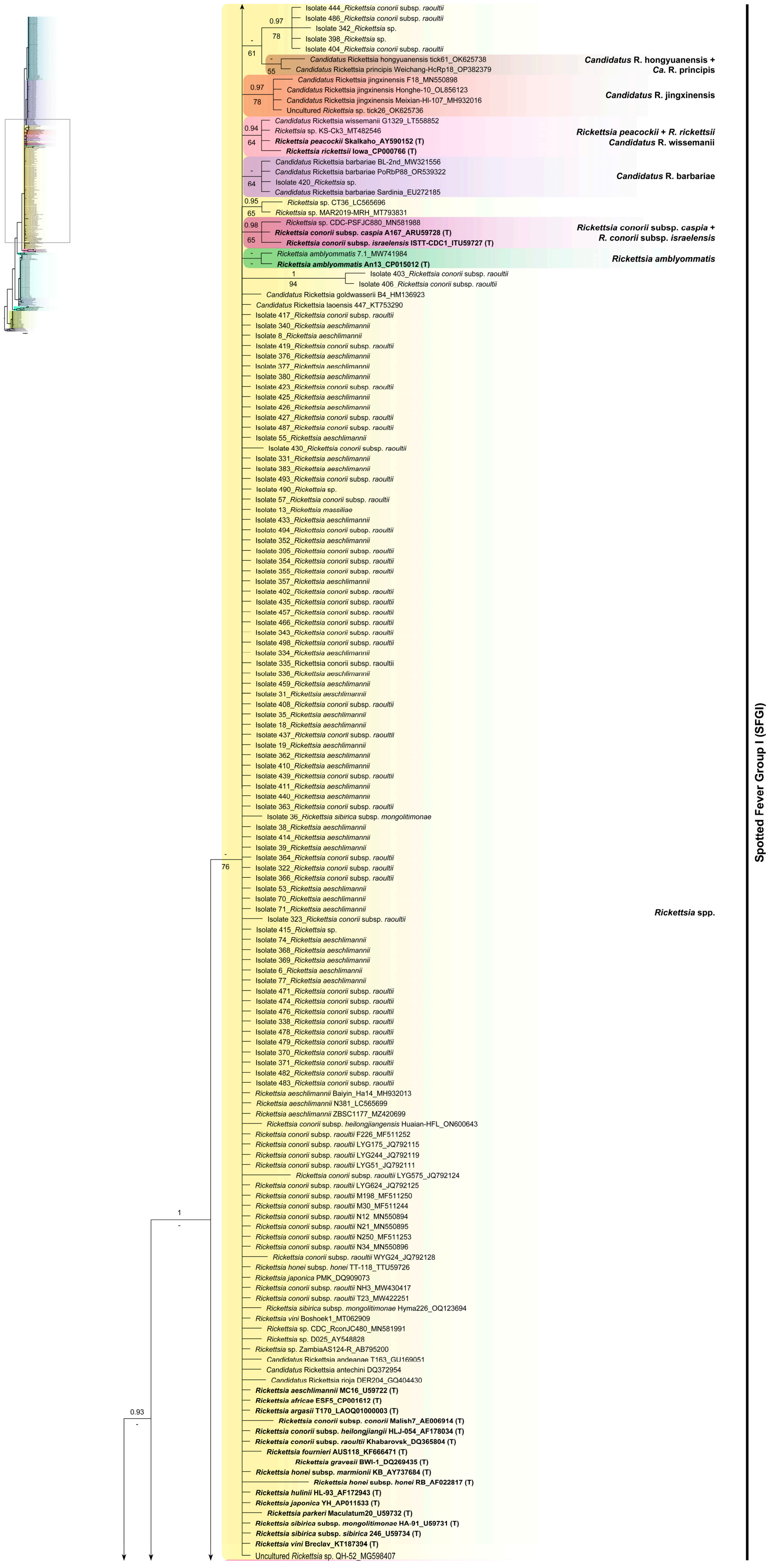

Figure S2. (Continued).

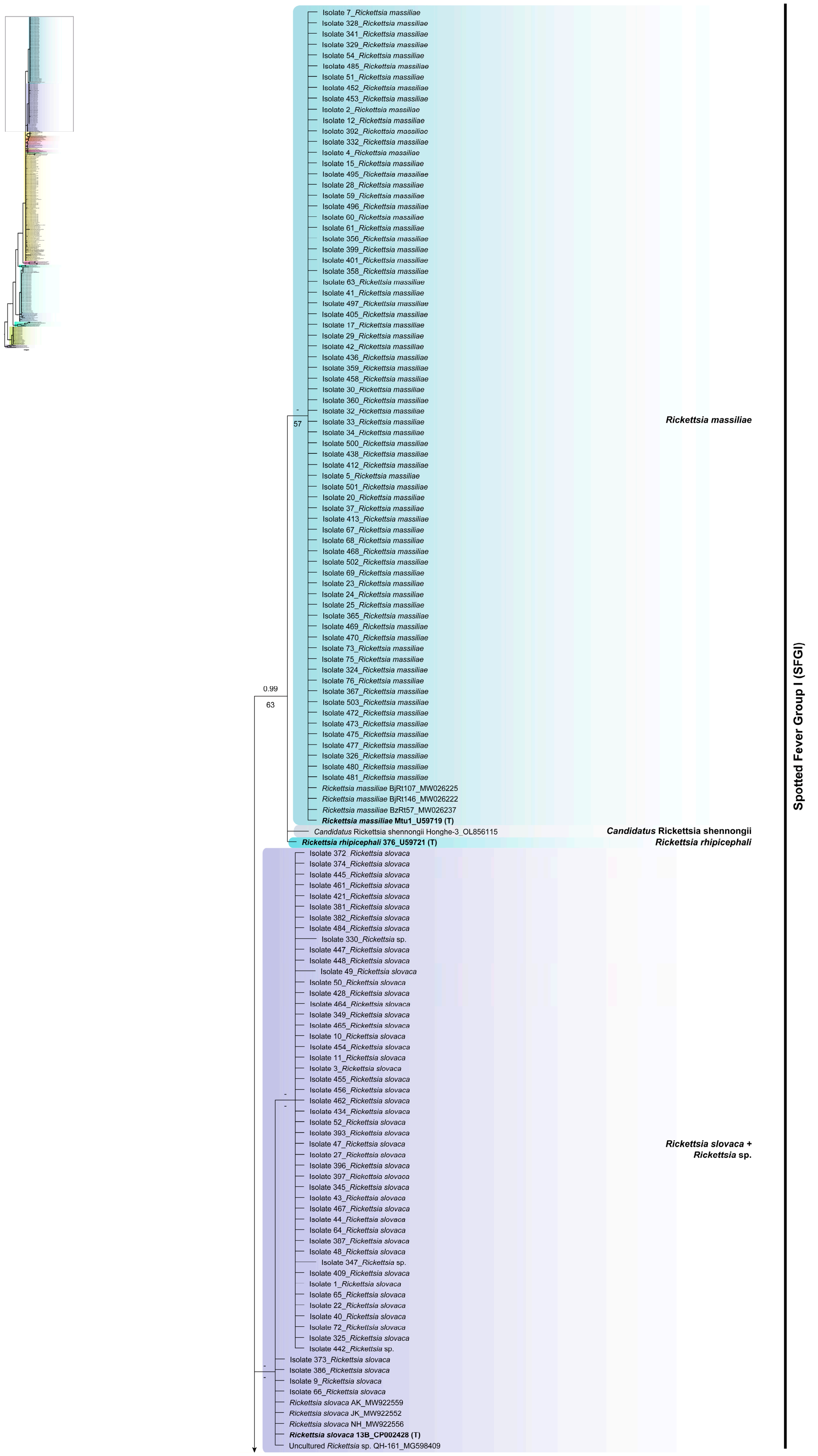

SUPPLEMENTARY TABLES

Table S1. Primers used for PCR amplification and size of the amplicons.

Target gene	Primer name	Primer sequence (5'–3')	Approximate amplicon size (bp)	Reference(s)
ompA	Rr190.70p	ATGGCGAATATTTCTCCAAAA	629–632	[20, 21]
	Rr190.701n	GTTCCGTTAATGGCAGCATCT		
gltA	RpCS.877p	GGGGGCCTGCTCACGGCGG	380–397	[21]
	RpCS.1258n	ATTGCAAAAAGTACAGTGAACA		

Table S2. Cycling parameters used in this study.

Gene/step		*ompA*		*gltA*	
		**Temp.**	**Time**	**Temp.**	**Time**
Initial denaturation		95 ºC	3 min	95 ºC	1 min
35 cycles	Denaturation	95 ºC	20 s	95 ºC	20 s
	Annealing	46 ºC	20 s	48 ºC	30 s
	Extension	63 ºC	1 min	60 ºC	2 min
Final extension		72 ºC	7 min	72 ºC	7 min

Table S3. Rickettsial isolates obtained from different tick species, and putative identity based on BLAST results of *ompA* and *gltA* partial sequences. Isolates whose BLAST-based identification varied depending on the gene analyzed are marked with an asterisk. Isolates with a doubtful identification based on *gltA* are marked with two asterisks.

Isolate number	Most similar GenBank accession				Putative species (*ompA*/*gltA*)
	ompA	Identity (%)	*gltA*	Identity (%)	
1	MK726320	100.00	MK652446	99.65	*R. slovaca*
2	KR401143	100.00	KY233220	99.65	*R. massiliae*
3	MK726320	99.18	MK652446	99.65	*R. slovaca*
4	KR401143	100.00	MG668822	100.00	*R. massiliae*
5	CP000683	99.81	KY233220	100.00	*R. massiliae*
6*	MF379306	100.00	OR496612	98.94	*R. aeschlimannii*/*R. conorii* subsp. *raoultii*
7	KR401143	98.98	MG668822	100.00	*R. massiliae*
8*	MK726330	97.01	MT178333	100.00	*R. aeschlimannii*/*R. conorii* subsp. *raoultii*
9	MF379297	100.00	KX506730	100.00	*R. slovaca*
10	MF379297	100.00	KY570487	99.64	*R. slovaca*
11	MF379297	99.82	KY570487	99.64	*R. slovaca*
12	CP000683	99.44	MG668822	100.00	*R. massiliae*
13*	CP000683	99.81	MT178333	100.00	*R. massiliae*/*R. conorii* subsp. *raoultii*
14	MF383610	100.00	ON640814	99.65	*R. monacensis*
15	CP000683	100.00	MG668822	100.00	*R. massiliae*
16	MF383610	97.77	ON640814	100.00	*R. monacensis*
17	CP003319	100.00	KY233220	100.00	*R. massiliae*
18	MK726330	99.82	MH932013	100.00	*R. aeschlimannii*
19	MK726330	100.00	MH932013	100.00	*R. aeschlimannii*
20	CP000683	100.00	MG668822	100.00	*R. massiliae*
21	MK203088	98.37	ON640814	100.00	*R. monacensis*
22	MK726320	98.39	KY570487	99.64	*R. slovaca*
23	CP000683	100.00	MG668822	99.64	*R. massiliae*
24	CP000683	100.00	MG668822	100.00	*R. massiliae*
25	CP003319	99.82	MG668822	100.00	*R. massiliae*
26	MK922659	99.23	ON640814	99.64	*R. monacensis*
27	MK726320	100.00	KY570487	99.64	*R. slovaca*
28	CP000683	99.45	KY233220	100.00	*R. massiliae*
29	CP000683	99.64	MG668822	100.00	*R. massiliae*
30	CP000683	97.53	MG668822	100.00	*R. massiliae*
31*	MK726330	100.00	MT178333	100.00	*R. aeschlimannii*/*R. conorii* subsp. *raoultii*
32	CP000683	100.00	MG668822	100.00	*R. massiliae*
33	CP003319	99.63	MG668822	100.00	*R. massiliae*
34	CP000683	100.00	MG668822	100.00	*R. massiliae*
35*	MF379306	100.00	MT178333	100.00	*R. aeschlimannii*/*R. conorii* subsp. *raoultii*
36*	KY513920	100.00	MG811708; CP040325; MH938655	100.00	*R. sibirica* subsp. *mongolitimonae*/ *R. sibirica* subsp. *sibirica*; *R. parkeri; R. africae*
37	CP000683	99.81	KY233220	99.60	*R. massiliae*
38	MF379306	99.81	MH932013	100.00	*R. aeschlimannii*
39	MF379306	100.00	MH932013	100.00	*R. aeschlimannii*
40	MF379297	100.00	KY570487	99.64	*R. slovaca*
41	KY379904	98.39	KY233220	97.29	*R. massiliae*
42	CP003319	99.61	KY233220	100.00	*R. massiliae*
43	MK726320	100.00	MK652446	99.65	*R. slovaca*
44	MK726320	100.00	MK652446	99.65	*R. slovaca*
45	MK922659	99.60	ON640814	100.00	*R. monacensis*
46	MF383610	100.00	ON640814	100.00	*R. monacensis*
47	MF379300	100.00	MK652446	99.65	*R. slovaca*
48	MF379295	100.00	MK652446	99.65	*R. slovaca*
49	MF379300	100.00	MK652446	99.65	*R. slovaca*
50	MF379297	100.00	MK652446	99.63	*R. slovaca*
51	CP000683	100.00	MG668822	100.00	*R. massiliae*
52	MF379297	99.82	KY570487	99.64	*R. slovaca*
53	MK726330	99.82	MH932013	100.00	*R. aeschlimannii*
54	CP000683	99.82	MG668822	100.00	*R. massiliae*

Table S3. (Continued).

55*	MK726330	99.81	MT178333	100.00	*R. aeschlimannii/R. conorii subsp. raoultii*
56	AH015165	100.00	KY213883	100.00	*R. monacensis*
57	AH015609	99.81	MT178333	100.00	*R. conorii* subsp. *raoultii*
58	MK203088	100.00	ON640814	100.00	*R. monacensis*
59	CP003319	99.81	MG668822	100.00	*R. massiliae*
60	CP000683	99.64	MG668822	100.00	*R. massiliae*
61	CP000683	99.64	MG668822	100.00	*R. massiliae*
62	MK922659	100.00	ON640814	100.00	*R. monacensis*
63	CP000683	100.00	KY233220	100.00	*R. massiliae*
64	MF379297	100.00	KY570487	99.63	*R. slovaca*
65	MK726320	100.00	MK652446	99.65	*R. slovaca*
66	MF379297	100.00	MK652446	100.00	*R. slovaca*
67	CP000683	99.82	MG668822	99.64	*R. massiliae*
68	CP003319	100.00	MG668822	100.00	*R. massiliae*
69	CP000683	99.82	AB872942	99.68	*R. massiliae*
70*	MF379306	99.82	MT178333	100.00	*R. aeschlimannii/R. conorii* subsp. *raoultii*
71*	MF379306	99.46	MT178333	100.00	*R. aeschlimannii/R. conorii* subsp. *raoultii*
72	MF379297	100.00	MK608656	99.68	*R. slovaca*
73	CP000683	100.00	AB872942	99.68	*R. massiliae*
74*	MF379306	99.82	MT178333	95.75	*R. aeschlimannii/R. conorii* subsp. *raoultii*
75	CP000683	100.00	AB872942	100.00	*R. massiliae*
76	CP000683	99.82	AB872942	100.00	*R. massiliae*
77*	MF379306	99.64	MT178333	100.00	*R. aeschlimannii/R. conorii* subsp. *raoultii*
78	MW817112	100.00	-	-	‘*Candidatus* R. rioja’
79	KR401143	100.00	-	-	*R. massiliae*
80	KR401143	100.00	-	-	*R. massiliae*
81	MF383610	99.56	-	-	*R. monacensis*
82	AH015609	99.81	-	-	*R. conorii* subsp. *raoultii*
83	CP003319	100.00	-	-	*R. massiliae*
84	MF379308	100.00	-	-	*R. aeschlimannii*
85	MK726330	99.26	-	-	*R. aeschlimannii*
86	MF379306	100.00	-	-	*R. aeschlimannii*
87	MF379306	99.26	-	-	*R. aeschlimannii*
88	MF383598	97.78	-	-	*R. conorii* subsp. *raoultii*
89	AH015609	100.00	-	-	*R. conorii* subsp. *raoultii*
90	MK922659	99.82	-	-	*R. monacensis*
91	MF383610	100.00	-	-	*R. monacensis*
92	MK726330	99.82	-	-	*R. aeschlimannii*
93	AH015609	100.00	-	-	*R. conorii* subsp. *raoultii*
94	MK922659	100.00	-	-	*R. monacensis*
95	MF383610	100.00	-	-	*R. monacensis*
96	MK726320	99.61	-	-	*R. slovaca*
97	AH015609	99.82	-	-	*R. conorii* subsp. *raoultii*
98	KR401143	100.00	-	-	*R. massiliae*
99	MK726330	100.00	-	-	*R. aeschlimannii*
100	MK726330	100.00	-	-	*R. aeschlimannii*
101	MK726320	100.00	-	-	*R. slovaca*
102	HM161773	100.00	-	-	*R. monacensis*
103	MK726330	100.00	-	-	*R. aeschlimannii*
104	MK726330	97.63	-	-	*R. aeschlimannii*
105	CP000683	99.44	-	-	*R. massiliae*
106	KR401143	100.00	-	-	*R. massiliae*
107	MF383610	99.62	-	-	*R. monacensis*
108	CP000683	100.00	-	-	*R. massiliae*
109	MF383610	99.61	-	-	*R. monacensis*
110	AH015609	99.81	-	-	*R. conorii* subsp. *raoultii*
111	JQ480842	99.41	-	-	*R. massiliae*
112	MK726320	99.60	-	-	*R. slovaca*
113	MK726330	100.00	-	-	*R. aeschlimannii*
114	MF383610	100.00	-	-	*R. monacensis*
115	MK726320	96.25	-	-	*R. slovaca*
116	MK726320	95.52	-	-	*R. slovaca*

Table S3. (Continued).

117	MK726320	97.99	-	-	*R. slovaca*
118	MF379295	100.00	-	-	*R. slovaca*
119	MW817112	100.00	-	-	'*Candidatus* R. rioja'
120	MK922659	99.63	-	-	*R. monacensis*
121	MK922659	100.00	-	-	*R. monacensis*
122	MK922659	99.82	-	-	*R. monacensis*
123	MF383610	100.00	-	-	*R. monacensis*
124	MK726320	100.00	-	-	*R. slovaca*
125	MF379295	100.00	-	-	*R. slovaca*
126	MK922659	100.00	-	-	*R. monacensis*
127	MF379295	100.00	-	-	*R. slovaca*
128	MK726320	95.91	-	-	*R. slovaca*
129	OQ403144	98.37	-	-	*R. aeschlimannii*
130	MF379306	100.00	-	-	*R. aeschlimannii*
131	CP000683	99.82	-	-	*R. massiliae*
132	MF379306	99.64	-	-	*R. aeschlimannii*
133	AH015609	100.00	-	-	*R. conorii* subsp. *raoultii*
134	OQ403144	99.39	-	-	*R. aeschlimannii*
135	MF379306	100.00	-	-	*R. aeschlimannii*
136	CP000683	100.00	-	-	*R. massiliae*
137	MF379306	100.00	-	-	*R. aeschlimannii*
138	MF379306	100.00	-	-	*R. aeschlimannii*
139	CP003319	99.81	-	-	*R. massiliae*
140	OQ403144	94.68	-	-	*R. aeschlimannii*
141	MG668829	97.01	-	-	'*Candidatus* R. barbariae'
142	MF383610	98.37	-	-	*R. monacensis*
143	AH015609	100.00	-	-	*R. conorii* subsp. *raoultii*
144	MF383598	96.07	-	-	*R. conorii* subsp. *raoultii*
145	MF383598	98.32	-	-	*R. conorii* subsp. *raoultii*
146	AH015609	99.82	-	-	*R. conorii* subsp. *raoultii*
147	CP000683	100.00	-	-	*R. massiliae*
148	JQ480842	99.81	-	-	*R. massiliae*
149	MK726330	100.00	-	-	*R. aeschlimannii*
150	CP000683	100.00	-	-	*R. massiliae*
151	CP000683	99.82	-	-	*R. massiliae*
152	MF379295	100.00	-	-	*R. slovaca*
153	MK922659	100.00	-	-	*R. monacensis*
154	AH015609	99.63	-	-	*R. conorii* subsp. *raoultii*
155	MF379306	99.82	-	-	*R. aeschlimannii*
156	MF379297	100.00	-	-	*R. slovaca*
157	MK726330	99.82	-	-	*R. aeschlimannii*
158	CP003319	99.80	-	-	*R. massiliae*
159	MF379306	99.82	-	-	*R. aeschlimannii*
160	MK726330	99.80	-	-	*R. aeschlimannii*
161	MF383610	99.62	-	-	*R. monacensis*
162	MK726320	100.00	-	-	*R. slovaca*
163	MF379306	99.82	-	-	*R. aeschlimannii*
164	MF383598	98.48	-	-	*R. conorii* subsp. *raoultii*
165	MF383610	100.00	-	-	*R. monacensis*
166	MK726320	100.00	-	-	*R. slovaca*
167	AH015609	99.44	-	-	*R. conorii* subsp. *raoultii*
168	MF383610	99.81	-	-	*R. monacensis*
169	MW817112	99.80	-	-	'*Candidatus* R. rioja'
170	HM161773	100.00	-	-	*R. monacensis*
171	MF379300	100.00	-	-	*R. slovaca*
172	MK922659	99.81	-	-	*R. monacensis*
173	HM161773	99.60	-	-	*R. monacensis*
174	MF379300	100.00	-	-	*R. slovaca*
175	MF379297	99.82	-	-	*R. slovaca*
176	MF383598	98.39	-	-	*R. conorii* subsp. *raoultii*
177	MF379297	100.00	-	-	*R. slovaca*
178	MF383598	98.65	-	-	*R. conorii* subsp. *raoultii*

Table S3. (Continued).

179	MF379297	100.00	-	-	*R. slovaca*
180	MF379306	100.00	-	-	*R. aeschlimannii*
181	MF379297	99.82	-	-	*R. slovaca*
182	MF379297	100.00	-	-	*R. slovaca*
183	CP003319	99.81	-	-	*R. massiliae*
184	MK922659	100.00	-	-	*R. monacensis*
185	CP003319	99.81	-	-	*R. massiliae*
186	MK726330	100.00	-	-	*R. aeschlimannii*
187	MK726320	100.00	-	-	*R. slovaca*
188	MF379306	100.00	-	-	*R. aeschlimannii*
189	MW817112	100.00	-	-	'*Candidatus* R. rioja'
190	AH015609	100.00	-	-	*R. conorii* subsp. *raoultii*
191	MW817112	100.00	-	-	'*Candidatus* R. rioja'
192	U43794	100.00	-	-	*R. conorii* subsp. *conorii*
193	U43794	100.00	-	-	*R. conorii* subsp. *conorii*
194	MF379306	100.00	-	-	*R. aeschlimannii*
195	CP003319	100.00	-	-	*R. massiliae*
196	CP003319	100.00	-	-	*R. massiliae*
197	MK726320	100.00	-	-	*R. slovaca*
198	MK726330	100.00	-	-	*R. aeschlimannii*
199	MF379306	99.81	-	-	*R. aeschlimannii*
200	MF379306	99.81	-	-	*R. aeschlimannii*
201	MF379306	100.00	-	-	*R. aeschlimannii*
202	MF383610	99.80	-	-	*R. monacensis*
203	MF383610	100.00	-	-	*R. monacensis*
204	AH015609	100.00	-	-	*R. conorii* subsp. *raoultii*
205	AH015609	99.59	-	-	*R. conorii* subsp. *raoultii*
206	MK726320	99.62	-	-	*R. slovaca*
207	MK726320	100.00	-	-	*R. slovaca*
208	MF383610	100.00	-	-	*R. monacensis*
209	MK726320	100.00	-	-	*R. slovaca*
210	MF383610	100.00	-	-	*R. monacensis*
211	MF383610	100.00	-	-	*R. monacensis*
212	MF383598	98.39	-	-	*R. conorii* subsp. *raoultii*
213	MK922659	100.00	-	-	*R. monacensis*
214	HM161773	100.00	-	-	*R. monacensis*
215	MK922659	100.00	-	-	*R. monacensis*
216	MK922659	100.00	-	-	*R. monacensis*
217	MK922659	99.81	-	-	*R. monacensis*
218	MW817112	99.60	-	-	'*Candidatus* R. rioja'
219	MF379300	100.00	-	-	*R. slovaca*
220	MW817112	99.80	-	-	'*Candidatus* R. rioja'
221	GQ404429	99.32	-	-	'*Candidatus* R. rioja'
222	MW817112	100.00	-	-	'*Candidatus* R. rioja'
223	MF379297	100.00	-	-	*R. slovaca*
224	KR401143	100.00	-	-	*R. massiliae*
225	GQ404429	99.79	-	-	'*Candidatus* R. rioja'
226	MF379300	100.00	-	-	*R. slovaca*
227	MF379306	99.25	-	-	*R. aeschlimannii*
228	MF379308	100.00	-	-	*R. aeschlimannii*
229	CP000683	100.00	-	-	*R. massiliae*
230	MK726330	100.00	-	-	*R. aeschlimannii*
231	CP000683	100.00	-	-	*R. massiliae*
232	HM161773	99.80	-	-	*R. monacensis*
233	KR401143	100.00	-	-	*R. massiliae*
234	MK726330	99.79	-	-	*R. aeschlimannii*
235	MF379306	99.81	-	-	*R. aeschlimannii*
236	MK726330	100.00	-	-	*R. aeschlimannii*
237	MK726330	100.00	-	-	*R. aeschlimannii*
238	JQ480842	100.00	-	-	*R. massiliae*
239	JQ480842	100.00	-	-	*R. massiliae*
240	CP000683	100.00	-	-	*R. massiliae*

Table S3. (Continued).

241	MF379308	100.00	-	-	*R. aeschlimannii*
242	MK726330	100.00	-	-	*R. aeschlimannii*
243	KY319224	98.72	-	-	*R. monacensis*
244	MF383610	100.00	-	-	*R. monacensis*
245	MF383610	100.00	-	-	*R. monacensis*
246	MF383610	100.00	-	-	*R. monacensis*
247	MF383610	100.00	-	-	*R. monacensis*
248	HM161773	99.80	-	-	*R. monacensis*
249	GQ404429	99.78	-	-	'*Candidatus* R. rioja'
250	MK726320	100.00	-	-	*R. slovaca*
251	HM161773	100.00	-	-	*R. monacensis*
252	MF383610	100.00	-	-	*R. monacensis*
253	MK726330	100.00	-	-	*R. aeschlimannii*
254	MF383610	100.00	-	-	*R. monacensis*
255	MK726320	99.81	-	-	*R. slovaca*
256	CP000683	99.81	-	-	*R. massiliae*
257	MF383610	100.00	-	-	*R. monacensis*
258	HM161773	99.53	-	-	*R. monacensis*
259	MF379297	100.00	-	-	*R. slovaca*
260	EF028201	99.60	-	-	'*Candidatus* R. rioja'
261	MF383610	98.96	-	-	*R. monacensis*
262	MF383610	100.00	-	-	*R. monacensis*
263	MF383610	100.00	-	-	*R. monacensis*
264	MF383610	100.00	-	-	*R. monacensis*
265	MF383610	100.00	-	-	*R. monacensis*
266	MF383610	100.00	-	-	*R. monacensis*
267	MF383610	100.00	-	-	*R. monacensis*
268	MF383610	99.78	-	-	*R. monacensis*
269	MF383610	100.00	-	-	*R. monacensis*
270	CP000683	100.00	-	-	*R. massiliae*
271	MW817117	98.60	-	-	'*Candidatus* R. rioja'
272	MF379297	100.00	-	-	*R. slovaca*
273	MF383598	98.10	-	-	*R. conorii* subsp. *raoultii*
274	MK726320	100.00	-	-	*R. slovaca*
275	MK922659	99.81	-	-	*R. monacensis*
276	MF383610	99.34	-	-	*R. monacensis*
277	MK922659	100.00	-	-	*R. monacensis*
278	CP000683	100.00	-	-	*R. massiliae*
279	CP000683	100.00	-	-	*R. massiliae*
280	MF383610	99.63	-	-	*R. monacensis*
281	CP000683	99.82	-	-	*R. massiliae*
282	MF383610	100.00	-	-	*R. monacensis*
283	MF383610	99.81	-	-	*R. monacensis*
284	AH015609	99.82	-	-	*R. conorii* subsp. *raoultii*
285	CP000683	100.00	-	-	*R. massiliae*
286	MF383610	99.63	-	-	*R. monacensis*
287	MF383610	100.00	-	-	*R. monacensis*
288	MK203088	100.00	-	-	*R. monacensis*
289	JQ480842	100.00	-	-	*R. massiliae*
290	KU723514	99.72	-	-	*R. conorii* subsp. *raoultii*
291	AH015609	98.89	-	-	*R. conorii* subsp. *raoultii*
292	CP000683	100.00	-	-	*R. massiliae*
293	MF379297	100.00	-	-	*R. slovaca*
294	CP000683	99.81	-	-	*R. massiliae*
295	CP000683	99.82	-	-	*R. massiliae*
296	CP000683	99.64	-	-	*R. massiliae*
297	CP000683	99.63	-	-	*R. massiliae*
298	MK726330	100.00	-	-	*R. aeschlimannii*
299	CP000683	99.82	-	-	*R. massiliae*
300	MF383598	97.89	-	-	*R. conorii* subsp. *raoultii*
301	MK726330	100.00	-	-	*R. aeschlimannii*
302	CP000683	100.00	-	-	*R. massiliae*

Table S3. (Continued).

303	CP003319	100.00	-	-	*R. massiliae*
304	CP000683	100.00	-	-	*R. massiliae*
305	MK922659	100.00	-	-	*R. monacensis*
306	CP000683	99.82	-	-	*R. massiliae*
307	CP000683	100.00	-	-	*R. massiliae*
308	KY233237	99.31	-	-	*R. massiliae*
309	MK726330	100.00	-	-	*R. aeschlimannii*
310	CP000683	100.00	-	-	*R. massiliae*
311	CP000683	99.28	-	-	*R. massiliae*
312	-	-	ON640814	100.00	*R. monacensis*
313	-	-	MG190375	100.00	*R. helvetica*
314	-	-	ON640814	100.00	*R. monacensis*
315	-	-	ON640814	100.00	*R. monacensis*
316	-	-	ON640814	100.00	*R. monacensis*
317	-	-	ON640814	100.00	*R. monacensis*
318	-	-	MH018971	100.00	*R. helvetica*
319	-	-	ON640814	100.00	*R. monacensis*
320	-	-	ON640814	100.00	*R. monacensis*
321	-	-	ON640814	100.00	*R. monacensis*
322	-	-	MT178333	100.00	*R. conorii* subsp. *raoultii*
323	-	-	MT178333	93.87	*R. conorii* subsp. *raoultii*
324	-	-	KY233220	100.00	*R. massiliae*
325	-	-	MK652446	99.65	*R. slovaca*
326	-	-	KY233220	100.00	*R. massiliae*
327	-	-	MH018971	100.00	*R. helvetica*
328	-	-	KY233220	100.00	*R. massiliae*
329	-	-	KY233220	100.00	*R. massiliae*
330**	-	-	OR420121;<br>OQ123695	98.77	*R. slovaca;*<br>*R. sibirica* subsp. *mongolitimonae*
331	-	-	MH932013	100.00	*R. aeschlimannii*
332	-	-	MG668822	100.00	*R. massiliae*
333	-	-	MH018971	100.00	*R. helvetica*
334	-	-	MH932013	100.00	*R. aeschlimannii*
335	-	-	MT178333	99.19	*R. conorii* subsp. *raoultii*
336	-	-	MH932013	100.00	*R. aeschlimannii*
337	-	-	KY213883	100.00	*R. monacensis*
338	-	-	MT178333	100.00	*R. conorii* subsp. *raoultii*
339	-	-	MG190375	100.00	*R. helvetica*
340	-	-	MH932013	99.29	*R. aeschlimannii*
341	-	-	KY233220	100.00	*R. massiliae*
342**	-	-	AY578114;<br>OK625738	97.55	'*Candidatus* R. principis';<br>'*Candidatus* R. hongyuanensis'
343	-	-	MT178333	100.00	*R. conorii* subsp. *raoultii*
344	-	-	ON640814	100.00	*R. monacensis*
345	-	-	KY570487	99.64	*R. slovaca*
346	-	-	ON640814	100.00	*R. monacensis*
347**	-	-	OR420121;<br>OQ123695	97.50	*R. slovaca*;<br>*R. sibirica* subsp. *mongolitimonae*
348	-	-	ON640814	100.00	*R. monacensis*
349	-	-	MK652446	99.65	*R. slovaca*
350	-	-	MG190375	100.00	*R. helvetica*
351	-	-	MH018971	100.00	*R. helvetica*
352	-	-	MH932013	100.00	*R. aeschlimannii*
353	-	-	ON640814	100.00	*R. monacensis*
354	-	-	MT178333	100.00	*R. conorii* subsp. *raoultii*
355	-	-	MT178333	100.00	*R. conorii* subsp. *raoultii*
356	-	-	KY233220	100.00	*R. massiliae*
357	-	-	MH932013	100.00	*R. aeschlimannii*
358	-	-	KY233220	100.00	*R. massiliae*
359	-	-	KY233220	100.00	*R. massiliae*
360	-	-	KY233220	100.00	*R. massiliae*
361	-	-	ON640814	100.00	*R. monacensis*
362	-	-	MH932013	100.00	*R. aeschlimannii*

Table S3. (Continued).

363	-	-	MT178333	100.00	*R. conorii subsp. raoultii*
364	-	-	MT178333	100.00	*R. conorii subsp. raoultii*
365	-	-	MG668822	100.00	*R. massiliae*
366	-	-	MT178333	100.00	*R. conorii subsp. raoultii*
367	-	-	KY233220	100.00	*R. massiliae*
368	-	-	MH932013	100.00	*R. aeschlimannii*
369	-	-	MH932013	100.00	*R. aeschlimannii*
370	-	-	MT178333	100.00	*R. conorii subsp. raoultii*
371	-	-	MT178333	100.00	*R. conorii subsp. raoultii*
372	-	-	MK652446	99.65	*R. slovaca*
373	-	-	MK652446	100.00	*R. slovaca*
374	-	-	MK652446	99.65	*R. slovaca*
375	-	-	MH018971	99.65	*R. helvetica*
376	-	-	MH932013	99.65	*R. aeschlimannii*
377	-	-	MH932013	99.65	*R. aeschlimannii*
378	-	-	ON640814	99.65	*R. monacensis*
379	-	-	ON640814	99.65	*R. monacensis*
380	-	-	MH932013	100.00	*R. aeschlimannii*
381	-	-	MK652446	99.65	*R. slovaca*
382	-	-	MK652446	99.65	*R. slovaca*
383	-	-	MH932013	100.00	*R. aeschlimannii*
384	-	-	ON640814	100.00	*R. monacensis*
385	-	-	ON640814	100.00	*R. monacensis*
386	-	-	MF383578	99.28	*R. slovaca*
387	-	-	MK652446	99.65	*R. slovaca*
388	-	-	ON640814	100.00	*R. monacensis*
389	-	-	ON640814	100.00	*R. monacensis*
390	-	-	ON640814	100.00	*R. monacensis*
391	-	-	ON640814	100.00	*R. monacensis*
392	-	-	MG668822	100.00	*R. massiliae*
393	-	-	MK652446	99.65	*R. slovaca*
394	-	-	ON640814	100.00	*R. monacensis*
395	-	-	MT178333	100.00	*R. conorii* subsp. *raoultii*
396	-	-	KY570487	99.64	*R. slovaca*
397	-	-	KY570487	99.64	*R. slovaca*
398**	-	-	AY578114; OK625738	92.31	‘*Candidatus* R. principis’; ‘*Candidatus* R. hongyuanensis’
399	-	-	KY233220	100.00	*R. massiliae*
400	-	-	ON640814	100.00	*R. monacensis*
401	-	-	KY233220	100.00	*R. massiliae*
402	-	-	MT178333	100.00	*R. conorii* subsp. *raoultii*
403	-	-	MT178333	97.10	*R. conorii* subsp. *raoultii*
404	-	-	OR496612	95.85	*R. conorii* subsp. *raoultii*
405	-	-	MG668822	100.00	*R. massiliae*
406	-	-	OQ789236	93.43	*R. conorii* subsp. *raoultii*
407	-	-	ON640814	100.00	*R. monacensis*
408	-	-	MT178333	100.00	*R. conorii* subsp. *raoultii*
409	-	-	MK652446	99.30	*R. slovaca*
410	-	-	MH932013	100.00	*R. aeschlimannii*
411	-	-	MH932013	100.00	*R. aeschlimannii*
412	-	-	KY233220	99.65	*R. massiliae*
413	-	-	MG668822	100.00	*R. massiliae*
414	-	-	MH932013	100.00	*R. aeschlimannii*
415**	-	-	OR288101; OR496612	100.00	*R. aeschlimannii*; *R. conorii* subsp. *raoultii*
416	-	-	MG190375	100.00	*R. helvetica*
417	-	-	MT178333	100.00	*R. conorii* subsp. *raoultii*
418	-	-	MH018981	100.00	*R. monacensis*
419	-	-	MT178333	99.64	*R. conorii* subsp. *raoultii*
420**	-	-	MK732478; MG668824; MG668826	99.64	*R. aeschlimannii*; *R. conorii* subsp. *raoultii*; ‘*Candidatus* R. barbariae’
421	-	-	KY570487	99.64	*R. slovaca*

Table S3. (Continued).

422	-	-	MH589997	96.64	*R. monacensis*
423	-	-	MT178333	99.28	*R. conorii subsp. raoultii*
424	-	-	ON640814	100.00	*R. monacensis*
425	-	-	MH932013	100.00	*R. aeschlimannii*
426	-	-	MH932013	100.00	*R. aeschlimannii*
427	-	-	MT178333	99.27	*R. conorii subsp. raoultii*
428	-	-	MK652446	99.65	*R. slovaca*
429	-	-	ON640814	100.00	*R. monacensis*
430	-	-	OQ789236	99.55	*R. conorii subsp. raoultii*
431	-	-	MG190375	100.00	*R. helvetica*
432	-	-	ON640814	100.00	*R. monacensis*
433	-	-	MH932013	100.00	*R. aeschlimannii*
434	-	-	KY570487	98.81	*R. slovaca*
435	-	-	MT178333	100.00	*R. conorii subsp. raoultii*
436	-	-	KY233220	100.00	*R. massiliae*
437	-	-	MT178333	100.00	*R. conorii subsp. raoultii*
438	-	-	MG668822	100.00	*R. massiliae*
439	-	-	MT178333	100.00	*R. conorii subsp. raoultii*
440	-	-	MH932013	100.00	*R. aeschlimannii*
441	-	-	MK792591	100.00	*R. monacensis*
442**	-	-	MK608656;<br>MH521292	99.28	*R. slovaca*;<br>*R. amblyommatis*
443	-	-	KY231196	100.00	*R. helvetica*
444	-	-	MT178333	97.46	*R. conorii* subsp. *raoultii*
445	-	-	KY570487	99.64	*R. slovaca*
446	-	-	MG190375	100.00	*R. helvetica*
447	-	-	KY570487	97.46	*R. slovaca*
448	-	-	MK652446	99.64	*R. slovaca*
449	-	-	ON640814	100.00	*R. monacensis*
450	-	-	ON640814	100.00	*R. monacensis*
451	-	-	ON640814	100.00	*R. monacensis*
452	-	-	MG668822	100.00	*R. massiliae*
453	-	-	KY233220	100.00	*R. massiliae*
454	-	-	MK652446	99.65	*R. slovaca*
455	-	-	KY570487	99.63	*R. slovaca*
456	-	-	KY570487	98.82	*R. slovaca*
457	-	-	MT178333	99.17	*R. conorii* subsp. *raoultii*
458	-	-	KY233220	100.00	*R. massiliae*
459	-	-	MH932013	100.00	*R. aeschlimannii*
460**	-	-	MH618386;<br>AP019563	100.00	*R. helvetica*;<br>*R. asiatica*
461	-	-	MK608656	99.67	*R. slovaca*
462	-	-	MK652446	99.65	*R. slovaca*
463	-	-	ON640814	100.00	*R. monacensis*
464	-	-	KY570487	99.59	*R. slovaca*
465	-	-	MK652446	99.65	*R. slovaca*
466	-	-	MT178333	100.00	*R. conorii* subsp. *raoultii*
467	-	-	MK652446	99.64	*R. slovaca*
468	-	-	KY233220	100.00	*R. massiliae*
469	-	-	KY233220	100.00	*R. massiliae*
470	-	-	KY233220	100.00	*R. massiliae*
471	-	-	MT178333	100.00	*R. conorii* subsp. *raoultii*
472	-	-	MG668822	100.00	*R. massiliae*
473	-	-	MG668822	100.00	*R. massiliae*
474	-	-	MT178333	100.00	*R. conorii* subsp. *raoultii*
475	-	-	MG668822	100.00	*R. massiliae*
476	-	-	MT178333	100.00	*R. conorii* subsp. *raoultii*
477	-	-	MG668822	100.00	*R. massiliae*
478	-	-	MT178333	100.00	*R. conorii* subsp. *raoultii*
479	-	-	MT178333	100.00	*R. conorii* subsp. *raoultii*
480	-	-	MG668822	100.00	*R. massiliae*
481	-	-	MG668822	100.00	*R. massiliae*

Table S3. (Continued).

482	-	-	MT178333	100.00	*R. conorii subsp. raoultii*
483	-	-	MT178333	100.00	*R. conorii subsp. raoultii*
484	-	-	MK652446	99.65	*R. slovaca*
485	-	-	MG668822	99.61	*R. massiliae*
486	-	-	MT178333	97.79	*R. conorii subsp. raoultii*
487	-	-	MT178333	100.00	*R. conorii subsp. raoultii*
488	-	-	MH018972	99.64	*R. helvetica*
489	-	-	ON640814	100.00	*R. monacensis*
490**	-	-	OR496612; OR539323	99.44	*R. conorii subsp. raoultii;* '*Candidatus* R. barbariae'
491	-	-	ON640814	100.00	*R. monacensis*
492	-	-	ON640814	100.00	*R. monacensis*
493	-	-	MT178333	100.00	*R. conorii subsp. raoultii*
494	-	-	MT178333	100.00	*R. conorii subsp. raoultii*
495	-	-	MG668822	98.79	*R. massiliae*
496	-	-	MG668822	100.00	*R. massiliae*
497	-	-	MG668822	100.00	*R. massiliae*
498	-	-	MT178333	100.00	*R. conorii subsp. raoultii*
499	-	-	MG190375	100.00	*R. helvetica*
500	-	-	KY233220	100.00	*R. massiliae*
501	-	-	KY233220	100.00	*R. massiliae*
502	-	-	MG668822	99.62	*R. massiliae*
503	-	-	KY233220	100.00	*R. massiliae*
504	-	-	ON640814	100.00	*R. monacensis*

Table S4. Rickettsial isolates newly obtained from different tick species in this study.

Isolate number	Host species	*Rickettsia* species	GenBank accession	
			ompA	***gltA***
1	*Dermacentor marginatus* (Sulzer, 1776)	*R. slovaca*	PP552066	PP552377
2	*Rhipicephalus bursa* (Canestrini and Fanzago, 1877)	*R. massiliae*	PP552067	PP552378
3	*Dermacentor marginatus* (Sulzer, 1776)	*R. slovaca*	PP552068	PP552379
4	*Rhipicephalus turanicus* (Pomerantsev, 1940)	*R. massiliae*	PP552069	PP552380
5	*Rhipicephalus bursa* (Canestrini and Fanzago, 1877)	*R. massiliae*	PP552070	PP552381
6	*Hyalomma marginatum* (Koch, 1844)	*R. aeschlimannii*	PP552071	PP552382
7	*Rhipicephalus sanguineus* (Latreille, 1806)	*R. massiliae*	PP552072	PP552383
8	*Hyalomma marginatum* (Koch, 1844)	*R. aeschlimannii*	PP552073	PP552384
9	*Dermacentor reticulatus* (Fabricius, 1794)	*R. slovaca*	PP552074	PP552385
10	*Dermacentor marginatus* (Sulzer, 1776)	*R. slovaca*	PP552075	PP552386
11	*Dermacentor marginatus* (Sulzer, 1776)	*R. slovaca*	PP552076	PP552387
12	*Rhipicephalus bursa* (Canestrini and Fanzago, 1877)	*R. massiliae*	PP552077	PP552388
13	*Dermacentor marginatus* (Sulzer, 1776)	*R. massiliae*	PP552078	PP552389
14	*Ixodes ricinus* (Linnaeus, 1758)	*R. monacensis*	PP552079	PP552390
15	*Rhipicephalus bursa* (Canestrini and Fanzago, 1877)	*R. massiliae*	PP552080	PP552391
16	*Ixodes ricinus* (Linnaeus, 1758)	*R. monacensis*	PP552081	PP552392
17	*Rhipicephalus* sp.	*R. massiliae*	PP552082	PP552393
18	*Hyalomma marginatum* (Koch, 1844)	*R. aeschlimannii*	PP552083	PP552394
19	*Hyalomma marginatum* (Koch, 1844)	*R. aeschlimannii*	PP552084	PP552395
20	*Rhipicephalus sanguineus* (Latreille, 1806)	*R. massiliae*	PP552085	PP552396
21	*Ixodes ricinus* (Linnaeus, 1758)	*R. monacensis*	PP552086	PP552397
22	*Dermacentor marginatus* (Sulzer, 1776)	*R. slovaca*	PP552087	PP552398
23	*Rhipicephalus turanicus* (Pomerantsev, 1940)	*R. massiliae*	PP552088	PP552399
24	*Rhipicephalus bursa* (Canestrini and Fanzago, 1877)	*R. massiliae*	PP552089	PP552400
25	*Rhipicephalus turanicus* (Pomerantsev, 1940)	*R. massiliae*	PP552090	PP552401
26	*Ixodes ricinus* (Linnaeus, 1758)	*R. monacensis*	PP552091	PP552402
27	*Dermacentor marginatus* (Sulzer, 1776)	*R. slovaca*	PP552092	PP552403
28	*Rhipicephalus turanicus* (Pomerantsev, 1940)	*R. massiliae*	PP552093	PP552404
29	*Rhipicephalus bursa* (Canestrini and Fanzago, 1877)	*R. massiliae*	PP552094	PP552405
30	*Rhipicephalus bursa* (Canestrini and Fanzago, 1877)	*R. massiliae*	PP552095	PP552406
31	*Hyalomma marginatum* (Koch, 1844)	*R. aeschlimannii*	PP552096	PP552407
32	*Rhipicephalus bursa* (Canestrini and Fanzago, 1877)	*R. massiliae*	PP552097	PP552408
33	*Rhipicephalus* sp.	*R. massiliae*	PP552098	PP552409
34	*Rhipicephalus bursa* (Canestrini and Fanzago, 1877)	*R. massiliae*	PP552099	PP552410
35	*Hyalomma marginatum* (Koch, 1844)	*R. aeschlimannii*	PP552100	PP552411
36	*Hyalomma marginatum* (Koch, 1844)	*R. sibirica* subsp. *mongolitimonae*	PP552101	PP552412
37	*Rhipicephalus* sp.	*R. massiliae*	PP552102	PP552413
38	*Hyalomma marginatum* (Koch, 1844)	*R. aeschlimannii*	PP552103	PP552414
39	*Hyalomma marginatum* (Koch, 1844)	*R. aeschlimannii*	PP552104	PP552415
40	*Dermacentor marginatus* (Sulzer, 1776)	*R. slovaca*	PP552105	PP552416
41	*Rhipicephalus sanguineus* (Latreille, 1806)	*R. massiliae*	PP552106	PP552417
42	*Rhipicephalus bursa* (Canestrini and Fanzago, 1877)	*R. massiliae*	PP552107	PP552418
43	*Dermacentor marginatus* (Sulzer, 1776)	*R. slovaca*	PP552108	PP552419
44	*Dermacentor marginatus* (Sulzer, 1776)	*R. slovaca*	PP552109	PP552420
45	*Ixodes ricinus* (Linnaeus, 1758)	*R. monacensis*	PP552110	PP552421
46	*Ixodes ricinus* (Linnaeus, 1758)	*R. monacensis*	PP552111	PP552422
47	*Dermacentor reticulatus* (Fabricius, 1794)	*R. slovaca*	PP552112	PP552423
48	*Dermacentor reticulatus* (Fabricius, 1794)	*R. slovaca*	PP552113	PP552424
49	*Dermacentor marginatus* (Sulzer, 1776)	*R. slovaca*	PP552114	PP552425
50	*Dermacentor marginatus* (Sulzer, 1776)	*R. slovaca*	PP552115	PP552426
51	*Rhipicephalus sanguineus* (Latreille, 1806)	*R. massiliae*	PP552116	PP552427
52	*Dermacentor marginatus* (Sulzer, 1776)	*R. slovaca*	PP552117	PP552428
53	*Hyalomma marginatum* (Koch, 1844)	*R. aeschlimannii*	PP552118	PP552429
54	*Rhipicephalus bursa* (Canestrini and Fanzago, 1877)	*R. massiliae*	PP552119	PP552430

Table S4. (Continued).

55	*Hyalomma marginatum* (Koch, 1844)	*R. aeschlimannii*	PP552120	PP552431
56	*Ixodes ricinus* (Linnaeus, 1758)	*R. monacensis*	PP552121	PP552432
57	*Dermacentor reticulatus* (Fabricius, 1794)	*R. conorii* subsp. *raoultii*	PP552122	PP552433
58	*Ixodes ricinus* (Linnaeus, 1758)	*R. monacensis*	PP552123	PP552434
59	*Rhipicephalus bursa* (Canestrini and Fanzago, 1877)	*R. massiliae*	PP552124	PP552435
60	*Rhipicephalus bursa* (Canestrini and Fanzago, 1877)	*R. massiliae*	PP552125	PP552436
61	*Rhipicephalus bursa* (Canestrini and Fanzago, 1877)	*R. massiliae*	PP552126	PP552437
62	*Ixodes ricinus* (Linnaeus, 1758)	*R. monacensis*	PP552127	PP552438
63	*Rhipicephalus bursa* (Canestrini and Fanzago, 1877)	*R. massiliae*	PP552128	PP552439
64	*Dermacentor marginatus* (Sulzer, 1776)	*R. slovaca*	PP552129	PP552440
65	*Dermacentor marginatus* (Sulzer, 1776)	*R. slovaca*	PP552130	PP552441
66	*Dermacentor marginatus* (Sulzer, 1776)	*R. slovaca*	PP552131	PP552442
67	*Rhipicephalus sanguineus* (Latreille, 1806)	*R. massiliae*	PP552132	PP552443
68	*Rhipicephalus sanguineus* (Latreille, 1806)	*R. massiliae*	PP552133	PP552444
69	*Rhipicephalus bursa* (Canestrini and Fanzago, 1877)	*R. massiliae*	PP552134	PP552445
70	*Hyalomma marginatum* (Koch, 1844)	*R. aeschlimannii*	PP552135	PP552446
71	*Hyalomma marginatum* (Koch, 1844)	*R. aeschlimannii*	PP552136	PP552447
72	*Dermacentor marginatus* (Sulzer, 1776)	*R. slovaca*	PP552137	PP552448
73	*Rhipicephalus bursa* (Canestrini and Fanzago, 1877)	*R. massiliae*	PP552138	PP552449
74	*Hyalomma marginatum* (Koch, 1844)	*R. aeschlimannii*	PP552139	PP552450
75	*Rhipicephalus sanguineus* (Latreille, 1806)	*R. massiliae*	PP552140	PP552451
76	*Rhipicephalus bursa* (Canestrini and Fanzago, 1877)	*R. massiliae*	PP552141	PP552452
77	*Hyalomma marginatum* (Koch, 1844)	*R. aeschlimannii*	PP552142	PP552453
78	*Dermacentor marginatus* (Sulzer, 1776)	'*Candidatus* R. rioja'	PP552143	-
79	*Rhipicephalus bursa* (Canestrini and Fanzago, 1877)	*R. massiliae*	PP552144	-
80	*Rhipicephalus sanguineus* (Latreille, 1806)	*R. massiliae*	PP552145	-
81	*Ixodes ricinus* (Linnaeus, 1758)	*R. monacensis*	PP552146	-
82	*Ixodes ricinus* (Linnaeus, 1758)	*R. conorii* subsp. *raoultii*	PP552147	-
83	*Rhipicephalus bursa* (Canestrini and Fanzago, 1877)	*R. massiliae*	PP552148	-
84	*Hyalomma marginatum* (Koch, 1844)	*R. aeschlimannii*	PP552149	-
85	*Ixodes ricinus* (Linnaeus, 1758)	*R. aeschlimannii*	PP552150	-
86	*Hyalomma lusitanicum* (Koch, 1844)	*R. aeschlimannii*	PP552151	-
87	*Hyalomma marginatum* (Koch, 1844)	*R. aeschlimannii*	PP552152	-
88	*Dermacentor marginatus* (Sulzer, 1776)	*R. conorii* subsp. *raoultii*	PP552153	-
89	*Dermacentor* sp.	*R. conorii* subsp. *raoultii*	PP552154	-
90	*Ixodes ricinus* (Linnaeus, 1758)	*R. monacensis*	PP552155	-
91	*Ixodes ricinus* (Linnaeus, 1758)	*R. monacensis*	PP552156	-
92	*Ixodes ricinus* (Linnaeus, 1758)	*R. aeschlimannii*	PP552157	-
93	*Ixodes ricinus* (Linnaeus, 1758)	*R. conorii* subsp. *raoultii*	PP552158	-
94	*Ixodes ricinus* (Linnaeus, 1758)	*R. monacensis*	PP552159	-
95	*Ixodes ricinus* (Linnaeus, 1758)	*R. monacensis*	PP552160	-
96	*Ixodes ricinus* (Linnaeus, 1758)	*R. slovaca*	PP552161	-
97	*Ixodes ricinus* (Linnaeus, 1758)	*R. conorii* subsp. *raoultii*	PP552162	-
98	*Rhipicephalus bursa* (Canestrini and Fanzago, 1877)	*R. massiliae*	PP552163	-
99	*Hyalomma marginatum* (Koch, 1844)	*R. aeschlimannii*	PP552164	-
100	*Hyalomma marginatum* (Koch, 1844)	*R. aeschlimannii*	PP552165	-
101	*Dermacentor marginatus* (Sulzer, 1776)	*R. slovaca*	PP552166	-
102	*Rhipicephalus turanicus* (Pomerantsev, 1940)	*R. monacensis*	PP552167	-
103	*Hyalomma lusitanicum* (Koch, 1844)	*R. aeschlimannii*	PP552168	-
104	*Rhipicephalus bursa* (Canestrini and Fanzago, 1877)	*R. aeschlimannii*	PP552169	-
105	*Rhipicephalus bursa* (Canestrini and Fanzago, 1877)	*R. massiliae*	PP552170	-
106	*Rhipicephalus turanicus* (Pomerantsev, 1940)	*R. massiliae*	PP552171	-
107	*Ixodes ricinus* (Linnaeus, 1758)	*R. monacensis*	PP552172	-
108	*Rhipicephalus bursa* (Canestrini and Fanzago, 1877)	*R. massiliae*	PP552173	-
109	*Rhipicephalus bursa* (Canestrini and Fanzago, 1877)	*R. monacensis*	PP552174	-
110	*Rhipicephalus bursa* (Canestrini and Fanzago, 1877)	*R. conorii* subsp. *raoultii*	PP552175	-
111	*Rhipicephalus bursa* (Canestrini and Fanzago, 1877)	*R. massiliae*	PP552176	-

Table S4. (Continued).

112	*Dermacentor marginatus* (Sulzer, 1776)	*R. slovaca*	PP552177	-
113	*Hyalomma marginatum* (Koch, 1844)	*R. aeschlimannii*	PP552178	-
114	*Ixodes ricinus* (Linnaeus, 1758)	*R. monacensis*	PP552179	-
115	*Ixodes ricinus* (Linnaeus, 1758)	*R. slovaca*	PP552180	-
116	*Ixodes ricinus* (Linnaeus, 1758)	*R. slovaca*	PP552181	-
117	*Rhipicephalus bursa* (Canestrini and Fanzago, 1877)	*R. slovaca*	PP552182	-
118	*Dermacentor marginatus* (Sulzer, 1776)	*R. slovaca*	PP552183	-
119	*Dermacentor marginatus* (Sulzer, 1776)	'*Candidatus* R. rioja'	PP552184	-
120	*Ixodes ricinus* (Linnaeus, 1758)	*R. monacensis*	PP552185	-
121	*Ixodes ricinus* (Linnaeus, 1758)	*R. monacensis*	PP552186	-
122	*Ixodes ricinus* (Linnaeus, 1758)	*R. monacensis*	PP552187	-
123	*Ixodes ricinus* (Linnaeus, 1758)	*R. monacensis*	PP552188	-
124	*Dermacentor marginatus* (Sulzer, 1776)	*R. slovaca*	PP552189	-
125	*Dermacentor marginatus* (Sulzer, 1776)	*R. slovaca*	PP552190	-
126	*Ixodes ricinus* (Linnaeus, 1758)	*R. monacensis*	PP552191	-
127	*Dermacentor marginatus* (Sulzer, 1776)	*R. slovaca*	PP552192	-
128	*Dermacentor marginatus* (Sulzer, 1776)	*R. slovaca*	PP552193	-
129	*Rhipicephalus bursa* (Canestrini and Fanzago, 1877)	*R. aeschlimannii*	PP552194	-
130	*Hyalomma lusitanicum* (Koch, 1844)	*R. aeschlimannii*	PP552195	-
131	*Rhipicephalus bursa* (Canestrini and Fanzago, 1877)	*R. massiliae*	PP552196	-
132	*Hyalomma lusitanicum* (Koch, 1844)	*R. aeschlimannii*	PP552197	-
133	*Haemaphysalis punctata* (Canestrini & Fanzago 1877)	*R. conorii* subsp. *raoultii*	PP552198	-
134	*Hyalomma marginatum* (Koch, 1844)	*R. aeschlimannii*	PP552199	-
135	*Hyalomma marginatum* (Koch, 1844)	*R. aeschlimannii*	PP552200	-
136	*Ixodes ricinus* (Linnaeus, 1758)	*R. massiliae*	PP552201	-
137	*Hyalomma marginatum* (Koch, 1844)	*R. aeschlimannii*	PP552202	-
138	*Hyalomma marginatum* (Koch, 1844)	*R. aeschlimannii*	PP552203	-
139	*Rhipicephalus sanguineus* (Latreille, 1806)	*R. massiliae*	PP552204	-
140	*Hyalomma marginatum* (Koch, 1844)	*R. aeschlimannii*	PP552205	-
141	*Rhipicephalus bursa* (Canestrini and Fanzago, 1877)	'*Candidatus* R. barbariae'	PP552206	-
142	*Ixodes ricinus* (Linnaeus, 1758)	*R. monacensis*	PP552207	-
143	*Dermacentor marginatus* (Sulzer, 1776)	*R. conorii* subsp. *raoultii*	PP552208	-
144	*Dermacentor marginatus* (Sulzer, 1776)	*R. conorii* subsp. *raoultii*	PP552209	-
145	*Dermacentor marginatus* (Sulzer, 1776)	'*Candidatus* R. rioja'	PP552210	-
146	*Dermacentor marginatus* (Sulzer, 1776)	*R. conorii* subsp. *raoultii*	PP552211	-
147	*Rhipicephalus bursa* (Canestrini and Fanzago, 1877)	*R. massiliae*	PP552212	-
148	*Rhipicephalus sanguineus* (Latreille, 1806)	*R. massiliae*	PP552213	-
149	*Hyalomma marginatum* (Koch, 1844)	*R. aeschlimannii*	PP552214	-
150	*Rhipicephalus bursa* (Canestrini and Fanzago, 1877)	*R. massiliae*	PP552215	-
151	*Rhipicephalus bursa* (Canestrini and Fanzago, 1877)	*R. massiliae*	PP552216	-
152	*Dermacentor marginatus* (Sulzer, 1776)	*R. slovaca*	PP552217	-
153	*Ixodes ricinus* (Linnaeus, 1758)	*R. monacensis*	PP552218	-
154	*Dermacentor marginatus* (Sulzer, 1776)	*R. conorii* subsp. *raoultii*	PP552219	-
155	*Hyalomma lusitanicum* (Koch, 1844)	*R. aeschlimannii*	PP552220	-
156	*Dermacentor marginatus* (Sulzer, 1776)	*R. slovaca*	PP552221	-
157	*Hyalomma marginatum* (Koch, 1844)	*R. aeschlimannii*	PP552222	-
158	*Rhipicephalus turanicus* (Pomerantsev, 1940)	*R. massiliae*	PP552223	-
159	*Ixodes ricinus* (Linnaeus, 1758)	*R. aeschlimannii*	PP552224	-
160	*Hyalomma marginatum* (Koch, 1844)	*R. aeschlimannii*	PP552225	-
161	*Ixodes ricinus* (Linnaeus, 1758)	*R. monacensis*	PP552226	-
162	*Dermacentor marginatus* (Sulzer, 1776)	*R. slovaca*	PP552227	-
163	*Hyalomma lusitanicum* (Koch, 1844)	*R. aeschlimannii*	PP552228	-
164	*Hyalomma lusitanicum* (Koch, 1844)	'*Candidatus* R. rioja'	PP552229	-
165	*Ixodes ricinus* (Linnaeus, 1758)	*R. monacensis*	PP552230	-
166	*Dermacentor marginatus* (Sulzer, 1776)	*R. slovaca*	PP552231	-

Table S4. (Continued).

167	*Dermacentor marginatus* (Sulzer, 1776)	*R. conorii subsp. raoultii*	PP552232	-
168	*Ixodes ricinus* (Linnaeus, 1758)	*R. monacensis*	PP552233	-
169	*Dermacentor marginatus* (Sulzer, 1776)	'*Candidatus* R. rioja'	PP552234	-
170	*Ixodes ricinus* (Linnaeus, 1758)	*R. monacensis*	PP552235	-
171	*Dermacentor marginatus* (Sulzer, 1776)	*R. slovaca*	PP552236	-
172	*Ixodes ricinus* (Linnaeus, 1758)	*R. monacensis*	PP552237	-
173	*Ixodes ricinus* (Linnaeus, 1758)	*R. monacensis*	PP552238	-
174	*Dermacentor marginatus* (Sulzer, 1776)	*R. slovaca*	PP552239	-
175	*Dermacentor reticulatus* (Fabricius, 1794)	*R. slovaca*	PP552240	-
176	*Dermacentor marginatus* (Sulzer, 1776)	'*Candidatus* R. rioja'	PP552241	-
177	*Dermacentor marginatus* (Sulzer, 1776)	*R. slovaca*	PP552242	-
178	*Dermacentor marginatus* (Sulzer, 1776)	'*Candidatus* R. rioja'	PP552243	-
179	*Dermacentor marginatus* (Sulzer, 1776)	*R. slovaca*	PP552244	-
180	*Ixodes ricinus* (Linnaeus, 1758)	*R. aeschlimannii*	PP552245	-
181	*Dermacentor marginatus* (Sulzer, 1776)	*R. slovaca*	PP552246	-
182	*Dermacentor reticulatus* (Fabricius, 1794)	*R. slovaca*	PP552247	-
183	*Rhipicephalus bursa* (Canestrini and Fanzago, 1877)	*R. massiliae*	PP552248	-
184	*Ixodes ricinus* (Linnaeus, 1758)	*R. monacensis*	PP552249	-
185	*Rhipicephalus bursa* (Canestrini and Fanzago, 1877)	*R. massiliae*	PP552250	-
186	*Hyalomma marginatum* (Koch, 1844)	*R. aeschlimannii*	PP552251	-
187	*Dermacentor marginatus* (Sulzer, 1776)	*R. slovaca*	PP552252	-
188	*Hyalomma lusitanicum* (Koch, 1844)	*R. aeschlimannii*	PP552253	-
189	*Dermacentor marginatus* (Sulzer, 1776)	'*Candidatus* R. rioja'	PP552254	-
190	*Dermacentor marginatus* (Sulzer, 1776)	*R. conorii* subsp. *raoultii*	PP552255	-
191	*Dermacentor marginatus* (Sulzer, 1776)	'*Candidatus* R. rioja'	PP552256	-
192	*Rhipicephalus bursa* (Canestrini and Fanzago, 1877)	*R. conorii* subsp. *conorii*	PP552257	-
193	*Rhipicephalus bursa* (Canestrini and Fanzago, 1877)	*R. conorii* subsp. *conorii*	PP552258	-
194	*Hyalomma marginatum* (Koch, 1844)	*R. aeschlimannii*	PP552259	-
195	*Rhipicephalus turanicus* (Pomerantsev, 1940)	*R. massiliae*	PP552260	-
196	*Rhipicephalus bursa* (Canestrini and Fanzago, 1877)	*R. massiliae*	PP552261	-
197	*Dermacentor marginatus* (Sulzer, 1776)	*R. slovaca*	PP552262	-
198	*Ixodes ricinus* (Linnaeus, 1758)	*R. aeschlimannii*	PP552263	-
199	*Hyalomma marginatum* (Koch, 1844)	*R. aeschlimannii*	PP552264	-
200	*Hyalomma marginatum* (Koch, 1844)	*R. aeschlimannii*	PP552265	-
201	*Hyalomma marginatum* (Koch, 1844)	*R. aeschlimannii*	PP552266	-
202	*Ixodes ricinus* (Linnaeus, 1758)	*R. monacensis*	PP552267	-
203	*Ixodes ricinus* (Linnaeus, 1758)	*R. monacensis*	PP552268	-
204	*Dermacentor reticulatus* (Fabricius, 1794)	*R. conorii* subsp. *raoultii*	PP552269	-
205	*Dermacentor reticulatus* (Fabricius, 1794)	*R. conorii* subsp. *raoultii*	PP552270	-
206	*Dermacentor marginatus* (Sulzer, 1776)	*R. slovaca*	PP552271	-
207	*Dermacentor marginatus* (Sulzer, 1776)	*R. slovaca*	PP552272	-
208	*Ixodes ricinus* (Linnaeus, 1758)	*R. monacensis*	PP552273	-
209	*Dermacentor marginatus* (Sulzer, 1776)	*R. slovaca*	PP552274	-
210	*Ixodes ricinus* (Linnaeus, 1758)	*R. monacensis*	PP552275	-
211	*Ixodes ricinus* (Linnaeus, 1758)	*R. monacensis*	PP552276	-
212	*Dermacentor marginatus* (Sulzer, 1776)	*R. conorii* subsp. *raoultii*	PP552277	-
213	*Ixodes ricinus* (Linnaeus, 1758)	*R. monacensis*	PP552278	-
214	*Ixodes ricinus* (Linnaeus, 1758)	*R. monacensis*	PP552279	-
215	*Ixodes ricinus* (Linnaeus, 1758)	*R. monacensis*	PP552280	-
216	*Ixodes ricinus* (Linnaeus, 1758)	*R. monacensis*	PP552281	-
217	*Ixodes ricinus* (Linnaeus, 1758)	*R. monacensis*	PP552282	-
218	*Dermacentor marginatus* (Sulzer, 1776)	'*Candidatus* R. rioja'	PP552283	-
219	*Dermacentor marginatus* (Sulzer, 1776)	*R. slovaca*	PP552284	-
220	*Dermacentor marginatus* (Sulzer, 1776)	'*Candidatus* R. rioja'	PP552285	-
221	*Dermacentor marginatus* (Sulzer, 1776)	'*Candidatus* R. rioja'	PP552286	-
222	*Dermacentor marginatus* (Sulzer, 1776)	'*Candidatus* R. rioja'	PP552287	-
223	*Dermacentor marginatus* (Sulzer, 1776)	*R. slovaca*	PP552288	-

Table S4. (Continued).

224	*Rhipicephalus bursa* (Canestrini and Fanzago, 1877)	*R. massiliae*	PP552289	-
225	*Dermacentor marginatus* (Sulzer, 1776)	'*Candidatus* R. rioja'	PP552290	-
226	*Dermacentor marginatus* (Sulzer, 1776)	*R. slovaca*	PP552291	-
227	*Hyalomma marginatum* (Koch, 1844)	*R. aeschlimannii*	PP552292	-
228	*Ixodes ricinus* (Linnaeus, 1758)	*R. aeschlimannii*	PP552293	-
229	*Rhipicephalus sanguineus* (Latreille, 1806)	*R. massiliae*	PP552294	-
230	*Hyalomma marginatum* (Koch, 1844)	*R. aeschlimannii*	PP552295	-
231	*Rhipicephalus bursa* (Canestrini and Fanzago, 1877)	*R. massiliae*	PP552296	-
232	*Ixodes ricinus* (Linnaeus, 1758)	*R. monacensis*	PP552297	-
233	*Rhipicephalus bursa* (Canestrini and Fanzago, 1877)	*R. massiliae*	PP552298	-
234	*Hyalomma marginatum* (Koch, 1844)	*R. aeschlimannii*	PP552299	-
235	*Hyalomma marginatum* (Koch, 1844)	*R. aeschlimannii*	PP552300	-
236	*Hyalomma marginatum* (Koch, 1844)	*R. aeschlimannii*	PP552301	-
237	*Hyalomma marginatum* (Koch, 1844)	*R. aeschlimannii*	PP552302	-
238	*Rhipicephalus bursa* (Canestrini and Fanzago, 1877)	*R. massiliae*	PP552303	-
239	*Rhipicephalus bursa* (Canestrini and Fanzago, 1877)	*R. massiliae*	PP552304	-
240	*Rhipicephalus bursa* (Canestrini and Fanzago, 1877)	*R. massiliae*	PP552305	-
241	*Hyalomma marginatum* (Koch, 1844)	*R. aeschlimannii*	PP552306	-
242	*Hyalomma marginatum* (Koch, 1844)	*R. aeschlimannii*	PP552307	-
243	*Ixodes ricinus* (Linnaeus, 1758)	*R. monacensis*	PP552308	-
244	*Ixodes ricinus* (Linnaeus, 1758)	*R. monacensis*	PP552309	-
245	*Ixodes ricinus* (Linnaeus, 1758)	*R. monacensis*	PP552310	-
246	*Ixodes ricinus* (Linnaeus, 1758)	*R. monacensis*	PP552311	-
247	*Ixodes ricinus* (Linnaeus, 1758)	*R. monacensis*	PP552312	-
248	*Ixodes ricinus* (Linnaeus, 1758)	*R. monacensis*	PP552313	-
249	*Dermacentor marginatus* (Sulzer, 1776)	'*Candidatus* R. rioja'	PP552314	-
250	Not available	*R. slovaca*	PP552315	-
251	*Ixodes ricinus* (Linnaeus, 1758)	*R. monacensis*	PP552316	-
252	*Ixodes ricinus* (Linnaeus, 1758)	*R. monacensis*	PP552317	-
253	*Hyalomma lusitanicum* (Koch, 1844)	*R. aeschlimannii*	PP552318	-
254	*Ixodes ricinus* (Linnaeus, 1758)	*R. monacensis*	PP552319	-
255	*Dermacentor marginatus* (Sulzer, 1776)	*R. slovaca*	PP552320	-
256	*Dermacentor marginatus* (Sulzer, 1776)	*R. massiliae*	PP552321	-
257	*Ixodes ricinus* (Linnaeus, 1758)	*R. monacensis*	PP552322	-
258	*Ixodes ricinus* (Linnaeus, 1758)	*R. monacensis*	PP552323	-
259	*Dermacentor marginatus* (Sulzer, 1776)	*R. slovaca*	PP552324	-
260	*Dermacentor marginatus* (Sulzer, 1776)	'*Candidatus* R. rioja'	PP552325	-
261	*Ixodes ricinus* (Linnaeus, 1758)	*R. monacensis*	PP552326	-
262	*Ixodes ricinus* (Linnaeus, 1758)	*R. monacensis*	PP552327	-
263	*Ixodes ricinus* (Linnaeus, 1758)	*R. monacensis*	PP552328	-
264	*Ixodes ricinus* (Linnaeus, 1758)	*R. monacensis*	PP552329	-
265	*Ixodes ricinus* (Linnaeus, 1758)	*R. monacensis*	PP552330	-
266	*Ixodes ricinus* (Linnaeus, 1758)	*R. monacensis*	PP552331	-
267	*Ixodes ricinus* (Linnaeus, 1758)	*R. monacensis*	PP552332	-
268	*Ixodes ricinus* (Linnaeus, 1758)	*R. monacensis*	PP552333	-
269	*Ixodes ricinus* (Linnaeus, 1758)	*R. monacensis*	PP552334	-
270	*Rhipicephalus sanguineus* (Latreille, 1806)	*R. massiliae*	PP552335	-
271	*Dermacentor marginatus* (Sulzer, 1776)	'*Candidatus* R. rioja'	PP552336	-
272	*Dermacentor marginatus* (Sulzer, 1776)	*R. slovaca*	PP552337	-
273	*Dermacentor marginatus* (Sulzer, 1776)	'*Candidatus* R. rioja'	PP552338	-
274	*Dermacentor marginatus* (Sulzer, 1776)	*R. slovaca*	PP552339	-
275	*Ixodes ricinus* (Linnaeus, 1758)	*R. monacensis*	PP552340	-
276	*Ixodes ricinus* (Linnaeus, 1758)	*R. monacensis*	PP552341	-
277	*Ixodes ricinus* (Linnaeus, 1758)	*R. monacensis*	PP552342	-
278	*Rhipicephalus sanguineus* (Latreille, 1806)	*R. massiliae*	PP552343	-
279	*Ixodes ricinus* (Linnaeus, 1758)	*R. massiliae*	PP552344	-
280	*Ixodes ricinus* (Linnaeus, 1758)	*R. monacensis*	PP552345	-

Table S4. (Continued).

281	*Rhipicephalus bursa* (Canestrini and Fanzago, 1877)	*R. massiliae*	PP552346	-
282	*Haemaphysalis punctata* (Canestrini & Fanzago 1877)	*R. monacensis*	PP552347	-
283	*Ixodes ricinus* (Linnaeus, 1758)	*R. monacensis*	PP552348	-
284	*Dermacentor marginatus* (Sulzer, 1776)	*R. conorii* subsp. *raoultii*	PP552349	-
285	*Rhipicephalus sanguineus* (Latreille, 1806)	*R. massiliae*	PP552350	-
286	*Ixodes ricinus* (Linnaeus, 1758)	*R. monacensis*	PP552351	-
287	*Ixodes ricinus* (Linnaeus, 1758)	*R. monacensis*	PP552352	-
288	*Ixodes ricinus* (Linnaeus, 1758)	*R. monacensis*	PP552353	-
289	*Rhipicephalus sanguineus* (Latreille, 1806)	*R. massiliae*	PP552354	-
290	*Dermacentor reticulatus* (Fabricius, 1794)	*R. conorii* subsp. *raoultii*	PP552355	-
291	*Dermacentor marginatus* (Sulzer, 1776)	*R. conorii* subsp. *raoultii*	PP552356	-
292	*Rhipicephalus sanguineus* (Latreille, 1806)	*R. massiliae*	PP552357	-
293	*Dermacentor marginatus* (Sulzer, 1776)	*R. slovaca*	PP552358	-
294	*Rhipicephalus bursa* (Canestrini and Fanzago, 1877)	*R. massiliae*	PP552359	-
295	*Rhipicephalus sanguineus* (Latreille, 1806)	*R. massiliae*	PP552360	-
296	*Rhipicephalus sanguineus* (Latreille, 1806)	*R. massiliae*	PP552361	-
297	*Rhipicephalus sanguineus* (Latreille, 1806)	*R. massiliae*	PP552362	-
298	*Hyalomma marginatum* (Koch, 1844)	*R. aeschlimannii*	PP552363	-
299	*Rhipicephalus sanguineus* (Latreille, 1806)	*R. massiliae*	PP552364	-
300	*Dermacentor marginatus* (Sulzer, 1776)	*R. conorii* subsp. *raoultii*	PP552365	-
301	*Hyalomma marginatum* (Koch, 1844)	*R. aeschlimannii*	PP552366	-
302	*Rhipicephalus bursa* (Canestrini and Fanzago, 1877)	*R. massiliae*	PP552367	-
303	*Haemaphysalis punctata* (Canestrini & Fanzago 1877)	*R. massiliae*	PP552368	-
304	*Rhipicephalus bursa* (Canestrini and Fanzago, 1877)	*R. massiliae*	PP552369	-
305	*Ixodes ricinus* (Linnaeus, 1758)	*R. monacensis*	PP552370	-
306	*Rhipicephalus sanguineus* (Latreille, 1806)	*R. massiliae*	PP552371	-
307	*Rhipicephalus sanguineus* (Latreille, 1806)	*R. massiliae*	PP552372	-
308	*Rhipicephalus bursa* (Canestrini and Fanzago, 1877)	*R. massiliae*	PP552373	-
309	*Hyalomma marginatum* (Koch, 1844)	*R. aeschlimannii*	PP552374	-
310	*Rhipicephalus sanguineus* (Latreille, 1806)	*R. massiliae*	PP552375	-
311	*Rhipicephalus sanguineus* (Latreille, 1806)	*R. massiliae*	PP552376	-
312	*Ixodes ricinus* (Linnaeus, 1758)	*R. monacensis*	-	PP552454
313	*Ixodes ricinus* (Linnaeus, 1758)	*R. helvetica*	-	PP552455
314	*Ixodes ricinus* (Linnaeus, 1758)	*R. monacensis*	-	PP552456
315	*Ixodes ricinus* (Linnaeus, 1758)	*R. monacensis*	-	PP552457
316	*Ixodes ricinus* (Linnaeus, 1758)	*R. monacensis*	-	PP552458
317	*Ixodes ricinus* (Linnaeus, 1758)	*R. monacensis*	-	PP552459
318	*Ixodes ricinus* (Linnaeus, 1758)	*R. helvetica*	-	PP552460
319	*Ixodes ricinus* (Linnaeus, 1758)	*R. monacensis*	-	PP552461
320	*Ixodes ricinus* (Linnaeus, 1758)	*R. monacensis*	-	PP552462
321	*Ixodes ricinus* (Linnaeus, 1758)	*R. monacensis*	-	PP552463
322	*Dermacentor reticulatus* (Fabricius, 1794)	*R. conorii* subsp. *raoultii*	-	PP552464
323	*Dermacentor marginatus* (Sulzer, 1776)	*R. conorii* subsp. *raoultii*	-	PP552465
324	*Rhipicephalus bursa* (Canestrini and Fanzago, 1877)	*R. massiliae*	-	PP552466
325	*Dermacentor marginatus* (Sulzer, 1776)	*R. slovaca*	-	PP552467
326	*Rhipicephalus turanicus* (Pomerantsev, 1940)	*R. massiliae*	-	PP552468
327	*Ixodes ricinus* (Linnaeus, 1758)	*R. helvetica*	-	PP552469
328	*Rhipicephalus turanicus* (Pomerantsev, 1940)	*R. massiliae*	-	PP552470
329	*Rhipicephalus turanicus* (Pomerantsev, 1940)	*R. massiliae*	-	PP552471
330	*Ixodes ricinus* (Linnaeus, 1758)	*Rickettsia* sp.	-	PP552472
331	*Dermacentor marginatus* (Sulzer, 1776)	*R. aeschlimannii*	-	PP552473
332	*Rhipicephalus sanguineus* (Latreille, 1806)	*R. massiliae*	-	PP552474
333	*Ixodes ricinus* (Linnaeus, 1758)	*R. helvetica*	-	PP552475
334	*Hyalomma marginatum* (Koch, 1844)	*R. aeschlimannii*	-	PP552476
335	*Hyalomma marginatum* (Koch, 1844)	*R. conorii* subsp. *raoultii*	-	PP552477

Table S4. (Continued).

336	*Rhipicephalus sanguineus* (Latreille, 1806)	*R. aeschlimannii*	-	PP552478
337	*Hyalomma marginatum* (Koch, 1844)	*R. monacensis*	-	PP552479
338	*Ixodes ricinus* (Linnaeus, 1758)	*R. conorii subsp. raoultii*	-	PP552480
339	*Ixodes ricinus* (Linnaeus, 1758)	*R. helvetica*	-	PP552481
340	*Haemaphysalis punctata* (Canestrini & Fanzago 1877)	*R. aeschlimannii*	-	PP552482
341	*Rhipicephalus bursa* (Canestrini and Fanzago, 1877)	*R. massiliae*	-	PP552483
342	*Hyalomma marginatum* (Koch, 1844)	*Rickettsia* sp.	-	PP552484
343	*Dermacentor marginatus* (Sulzer, 1776)	*R. conorii* subsp. *raoultii*	-	PP552485
344	*Dermacentor reticulatus* (Fabricius, 1794)	*R. monacensis*	-	PP552486
345	*Ixodes ricinus* (Linnaeus, 1758)	*R. slovaca*	-	PP552487
346	*Ixodes ricinus* (Linnaeus, 1758)	*R. monacensis*	-	PP552488
347	*Dermacentor marginatus* (Sulzer, 1776)	*Rickettsia* sp.	-	PP552489
348	*Ixodes ricinus* (Linnaeus, 1758)	*R. monacensis*	-	PP552490
349	*Dermacentor marginatus* (Sulzer, 1776)	*R. slovaca*	-	PP552491
350	*Ixodes ricinus* (Linnaeus, 1758)	*R. helvetica*	-	PP552492
351	*Ixodes ricinus* (Linnaeus, 1758)	*R. helvetica*	-	PP552493
352	*Hyalomma marginatum* (Koch, 1844)	*R. aeschlimannii*	-	PP552494
353	*Ixodes ricinus* (Linnaeus, 1758)	*R. monacensis*	-	PP552495
354	*Dermacentor reticulatus* (Fabricius, 1794)	*R. conorii* subsp. *raoultii*	-	PP552496
355	*Ixodes ricinus* (Linnaeus, 1758)	*R. conorii* subsp. *raoultii*	-	PP552497
356	*Rhipicephalus turanicus* (Pomerantsev, 1940)	*R. massiliae*	-	PP552498
357	*Rhipicephalus sanguineus* (Latreille, 1806)	*R. aeschlimannii*	-	PP552499
358	*Rhipicephalus bursa* (Canestrini and Fanzago, 1877)	*R. massiliae*	-	PP552500
359	*Rhipicephalus* sp.	*R. massiliae*	-	PP552501
360	*Rhipicephalus* sp.	*R. massiliae*	-	PP552502
361	*Ixodes ricinus* (Linnaeus, 1758)	*R. monacensis*	-	PP552503
362	*Rhipicephalus bursa* (Canestrini and Fanzago, 1877)	*R. aeschlimannii*	-	PP552504
363	*Hyalomma marginatum* (Koch, 1844)	*R. conorii* subsp. *raoultii*	-	PP552505
364	*Hyalomma marginatum* (Koch, 1844)	*R. conorii* subsp. *raoultii*	-	PP552506
365	*Rhipicephalus turanicus* (Pomerantsev, 1940)	*R. massiliae*	-	PP552507
366	*Hyalomma marginatum* (Koch, 1844)	*R. conorii* subsp. *raoultii*	-	PP552508
367	*Rhipicephalus sanguineus* (Latreille, 1806)	*R. massiliae*	-	PP552509
368	*Ixodes ricinus* (Linnaeus, 1758)	*R. aeschlimannii*	-	PP552510
369	*Hyalomma lusitanicum* (Koch, 1844)	*R. aeschlimannii*	-	PP552511
370	Not available	*R. conorii* subsp. *raoultii*	-	PP552512
371	*Hyalomma marginatum* (Koch, 1844)	*R. conorii* subsp. *raoultii*	-	PP552513
372	*Dermacentor marginatus* (Sulzer, 1776)	*R. slovaca*	-	PP552514
373	*Dermacentor marginatus* (Sulzer, 1776)	*R. slovaca*	-	PP552515
374	*Dermacentor reticulatus* (Fabricius, 1794)	*R. slovaca*	-	PP552516
375	*Ixodes ricinus* (Linnaeus, 1758)	*R. helvetica*	-	PP552517
376	*Hyalomma marginatum* (Koch, 1844)	*R. aeschlimannii*	-	PP552518
377	*Hyalomma marginatum* (Koch, 1844)	*R. aeschlimannii*	-	PP552519
378	*Ixodes ricinus* (Linnaeus, 1758)	*R. monacensis*	-	PP552520
379	*Ixodes ricinus* (Linnaeus, 1758)	*R. monacensis*	-	PP552521
380	*Ixodes ricinus* (Linnaeus, 1758)	*R. aeschlimannii*	-	PP552522
381	*Dermacentor marginatus* (Sulzer, 1776)	*R. slovaca*	-	PP552523
382	*Dermacentor marginatus* (Sulzer, 1776)	*R. slovaca*	-	PP552524
383	Not available	*R. aeschlimannii*	-	PP552525
384	*Ixodes ricinus* (Linnaeus, 1758)	*R. monacensis*	-	PP552526
385	*Ixodes ricinus* (Linnaeus, 1758)	*R. monacensis*	-	PP552527
386	*Dermacentor marginatus* (Sulzer, 1776)	*R. slovaca*	-	PP552528
387	*Dermacentor marginatus* (Sulzer, 1776)	*R. slovaca*	-	PP552529
388	*Ixodes ricinus* (Linnaeus, 1758)	*R. monacensis*	-	PP552530
389	*Ixodes ricinus* (Linnaeus, 1758)	*R. monacensis*	-	PP552531
390	*Ixodes ricinus* (Linnaeus, 1758)	*R. monacensis*	-	PP552532
391	*Ixodes ricinus* (Linnaeus, 1758)	*R. monacensis*	-	PP552533

Table S4. (Continued).

392	*Rhipicephalus bursa* (Canestrini and Fanzago, 1877)	*R. massiliae*	-	PP552534
393	*Dermacentor marginatus* (Sulzer, 1776)	*R. slovaca*	-	PP552535
394	*Ixodes ricinus* (Linnaeus, 1758)	*R. monacensis*	-	PP552536
395	*Hyalomma marginatum* (Koch, 1844)	*R. conorii subsp. raoultii*	-	PP552537
396	*Dermacentor marginatus* (Sulzer, 1776)	*R. slovaca*	-	PP552538
397	*Dermacentor marginatus* (Sulzer, 1776)	*R. slovaca*	-	PP552539
398	*Dermacentor marginatus* (Sulzer, 1776)	*Rickettsia* sp.	-	PP552540
399	*Rhipicephalus sanguineus* (Latreille, 1806)	*R. massiliae*	-	PP552541
400	*Ixodes ricinus* (Linnaeus, 1758)	*R. monacensis*	-	PP552542
401	*Rhipicephalus bursa* (Canestrini and Fanzago, 1877)	*R. massiliae*	-	PP552543
402	*Ixodes ricinus* (Linnaeus, 1758)	*R. conorii* subsp. *raoultii*	-	PP552544
403	*Dermacentor marginatus* (Sulzer, 1776)	*R. conorii* subsp. *raoultii*	-	PP552545
404	*Dermacentor marginatus* (Sulzer, 1776)	*R. conorii* subsp. *raoultii*	-	PP552546
405	*Dermacentor marginatus* (Sulzer, 1776)	*R. massiliae*	-	PP552547
406	*Dermacentor marginatus* (Sulzer, 1776)	*R. conorii* subsp. *raoultii*	-	PP552548
407	*Ixodes ricinus* (Linnaeus, 1758)	*R. monacensis*	-	PP552549
408	*Hyalomma lusitanicum* (Koch, 1844)	*R. conorii* subsp. *raoultii*	-	PP552550
409	*Dermacentor marginatus* (Sulzer, 1776)	*R. slovaca*	-	PP552551
410	*Hyalomma lusitanicum* (Koch, 1844)	*R. aeschlimannii*	-	PP552552
411	*Hyalomma lusitanicum* (Koch, 1844)	*R. aeschlimannii*	-	PP552553
412	*Rhipicephalus sanguineus* (Latreille, 1806)	*R. massiliae*	-	PP552554
413	*Rhipicephalus sanguineus* (Latreille, 1806)	*R. massiliae*	-	PP552555
414	*Ixodes ricinus* (Linnaeus, 1758)	*R. aeschlimannii*	-	PP552556
415	*Hyalomma marginatum* (Koch, 1844)	*Rickettsia* sp.	-	PP552557
416	*Rhipicephalus bursa* (Canestrini and Fanzago, 1877)	*R. helvetica*	-	PP552558
417	*Hyalomma lusitanicum* (Koch, 1844)	*R. conorii* subsp. *raoultii*	-	PP552559
418	*Ixodes ricinus* (Linnaeus, 1758)	*R. monacensis*	-	PP552560
419	*Rhipicephalus bursa* (Canestrini and Fanzago, 1877)	*R. conorii* subsp. *raoultii*	-	PP552561
420	*Rhipicephalus bursa* (Canestrini and Fanzago, 1877)	*Rickettsia* sp.	-	PP552562
421	*Dermacentor marginatus* (Sulzer, 1776)	*R. slovaca*	-	PP552563
422	*Ixodes ricinus* (Linnaeus, 1758)	*R. monacensis*	-	PP552564
423	*Dermacentor marginatus* (Sulzer, 1776)	*R. conorii* subsp. *raoultii*	-	PP552565
424	*Ixodes ricinus* (Linnaeus, 1758)	*R. monacensis*	-	PP552566
425	*Hyalomma marginatum* (Koch, 1844)	*R. aeschlimannii*	-	PP552567
426	*Rhipicephalus bursa* (Canestrini and Fanzago, 1877)	*R. aeschlimannii*	-	PP552568
427	*Dermacentor marginatus* (Sulzer, 1776)	*R. conorii* subsp. *raoultii*	-	PP552569
428	*Dermacentor marginatus* (Sulzer, 1776)	*R. slovaca*	-	PP552570
429	*Ixodes ricinus* (Linnaeus, 1758)	*R. monacensis*	-	PP552571
430	*Dermacentor marginatus* (Sulzer, 1776)	*R. conorii* subsp. *raoultii*	-	PP552572
431	*Ixodes ricinus* (Linnaeus, 1758)	*R. helvetica*	-	PP552573
432	*Ixodes ricinus* (Linnaeus, 1758)	*R. monacensis*	-	PP552574
433	*Hyalomma lusitanicum* (Koch, 1844)	*R. aeschlimannii*	-	PP552575
434	*Dermacentor marginatus* (Sulzer, 1776)	*R. slovaca*	-	PP552576
435	*Ixodes ricinus* (Linnaeus, 1758)	*R. conorii* subsp. *raoultii*	-	PP552577
436	*Ixodes ricinus* (Linnaeus, 1758)	*R. massiliae*	-	PP552578
437	*Hyalomma marginatum* (Koch, 1844)	*R. conorii* subsp. *raoultii*	-	PP552579
438	*Ixodes ricinus* (Linnaeus, 1758)	*R. massiliae*	-	PP552580
439	*Hyalomma marginatum* (Koch, 1844)	*R. conorii* subsp. *raoultii*	-	PP552581
440	*Rhipicephalus bursa* (Canestrini and Fanzago, 1877)	*R. aeschlimannii*	-	PP552582
441	*Ixodes ricinus* (Linnaeus, 1758)	*R. monacensis*	-	PP552583
442	*Ixodes ricinus* (Linnaeus, 1758)	*Rickettsia* sp.	-	PP552584
443	*Ixodes ricinus* (Linnaeus, 1758)	*R. helvetica*	-	PP552585
444	*Dermacentor marginatus* (Sulzer, 1776)	*R. conorii* subsp. *raoultii*	-	PP552586
445	*Dermacentor marginatus* (Sulzer, 1776)	*R. slovaca*	-	PP552587
446	*Ixodes ricinus* (Linnaeus, 1758)	*R. helvetica*	-	PP552588
447	*Dermacentor marginatus* (Sulzer, 1776)	*R. slovaca*	-	PP552589
448	*Rhipicephalus bursa* (Canestrini and Fanzago, 1877)	*R. slovaca*	-	PP552590

Table S4. (Continued).

449	*Ixodes ricinus* (Linnaeus, 1758)	*R. monacensis*	-	PP552591
450	*Ixodes ricinus* (Linnaeus, 1758)	*R. monacensis*	-	PP552592
451	*Ixodes ricinus* (Linnaeus, 1758)	*R. monacensis*	-	PP552593
452	*Rhipicephalus bursa* (Canestrini and Fanzago, 1877)	*R. massiliae*	-	PP552594
453	*Rhipicephalus bursa* (Canestrini and Fanzago, 1877)	*R. massiliae*	-	PP552595
454	*Dermacentor reticulatus* (Fabricius, 1794)	*R. slovaca*	-	PP552596
455	*Dermacentor marginatus* (Sulzer, 1776)	*R. slovaca*	-	PP552597
456	*Dermacentor marginatus* (Sulzer, 1776)	*R. slovaca*	-	PP552598
457	*Dermacentor marginatus* (Sulzer, 1776)	*R. conorii* subsp. *raoultii*	-	PP552599
458	*Ixodes ricinus* (Linnaeus, 1758)	*R. massiliae*	-	PP552600
459	*Hyalomma marginatum* (Koch, 1844)	*R. aeschlimannii*	-	PP552601
460	*Ixodes ricinus* (Linnaeus, 1758)	*Rickettsia* sp.	-	PP552602
461	*Hyalomma marginatum* (Koch, 1844)	*R. slovaca*	-	PP552603
462	*Dermacentor marginatus* (Sulzer, 1776)	*R. slovaca*	-	PP552604
463	*Ixodes ricinus* (Linnaeus, 1758)	*R. monacensis*	-	PP552605
464	*Dermacentor marginatus* (Sulzer, 1776)	*R. slovaca*	-	PP552606
465	*Dermacentor marginatus* (Sulzer, 1776)	*R. slovaca*	-	PP552607
466	*Dermacentor reticulatus* (Fabricius, 1794)	*R. conorii* subsp. *raoultii*	-	PP552608
467	*Dermacentor reticulatus* (Fabricius, 1794)	*R. slovaca*	-	PP552609
468	*Rhipicephalus bursa* (Canestrini and Fanzago, 1877)	*R. massiliae*	-	PP552610
469	*Rhipicephalus bursa* (Canestrini and Fanzago, 1877)	*R. massiliae*	-	PP552611
470	*Rhipicephalus bursa* (Canestrini and Fanzago, 1877)	*R. massiliae*	-	PP552612
471	*Hyalomma marginatum* (Koch, 1844)	*R. conorii* subsp. *raoultii*	-	PP552613
472	*Rhipicephalus bursa* (Canestrini and Fanzago, 1877)	*R. massiliae*	-	PP552614
473	*Rhipicephalus bursa* (Canestrini and Fanzago, 1877)	*R. massiliae*	-	PP552615
474	*Hyalomma marginatum* (Koch, 1844)	*R. conorii* subsp. *raoultii*	-	PP552616
475	*Rhipicephalus bursa* (Canestrini and Fanzago, 1877)	*R. massiliae*	-	PP552617
476	*Hyalomma marginatum* (Koch, 1844)	*R. conorii* subsp. *raoultii*	-	PP552618
477	*Rhipicephalus bursa* (Canestrini and Fanzago, 1877)	*R. massiliae*	-	PP552619
478	*Hyalomma lusitanicum* (Koch, 1844)	*R. conorii* subsp. *raoultii*	-	PP552620
479	*Hyalomma marginatum* (Koch, 1844)	*R. conorii* subsp. *raoultii*	-	PP552621
480	*Rhipicephalus bursa* (Canestrini and Fanzago, 1877)	*R. massiliae*	-	PP552622
481	*Rhipicephalus bursa* (Canestrini and Fanzago, 1877)	*R. massiliae*	-	PP552623
482	*Hyalomma marginatum* (Koch, 1844)	*R. conorii* subsp. *raoultii*	-	PP552624
483	*Hyalomma marginatum* (Koch, 1844)	*R. conorii* subsp. *raoultii*	-	PP552625
484	*Hyalomma marginatum* (Koch, 1844)	*R. slovaca*	-	PP552626
485	*Rhipicephalus sanguineus* (Latreille, 1806)	*R. massiliae*	-	PP552627
486	*Dermacentor reticulatus* (Fabricius, 1794)	*R. conorii* subsp. *raoultii*	-	PP552628
487	*Hyalomma marginatum* (Koch, 1844)	*R. conorii* subsp. *raoultii*	-	PP552629
488	*Ixodes ricinus* (Linnaeus, 1758)	*R. helvetica*	-	PP552630
489	*Ixodes ricinus* (Linnaeus, 1758)	*R. monacensis*	-	PP552631
490	*Dermacentor reticulatus* (Fabricius, 1794)	*Rickettsia* sp.	-	PP552632
491	*Ixodes ricinus* (Linnaeus, 1758)	*R. monacensis*	-	PP552633
492	*Ixodes ricinus* (Linnaeus, 1758)	*R. monacensis*	-	PP552634
493	*Dermacentor reticulatus* (Fabricius, 1794)	*R. conorii* subsp. *raoultii*	-	PP552635
494	*Dermacentor marginatus* (Sulzer, 1776)	*R. conorii* subsp. *raoultii*	-	PP552636
495	*Rhipicephalus bursa* (Canestrini and Fanzago, 1877)	*R. massiliae*	-	PP552637
496	*Rhipicephalus sanguineus* (Latreille, 1806)	*R. massiliae*	-	PP552638
497	*Rhipicephalus sanguineus* (Latreille, 1806)	*R. massiliae*	-	PP552639
498	*Hyalomma marginatum* (Koch, 1844)	*R. conorii* subsp. *raoultii*	-	PP552640
499	*Ixodes ricinus* (Linnaeus, 1758)	*R. helvetica*	-	PP552641
500	*Rhipicephalus bursa* (Canestrini and Fanzago, 1877)	*R. massiliae*	-	PP552642
501	*Rhipicephalus bursa* (Canestrini and Fanzago, 1877)	*R. massiliae*	-	PP552643
502	*Rhipicephalus sanguineus* (Latreille, 1806)	*R. massiliae*	-	PP552644
503	*Rhipicephalus bursa* (Canestrini and Fanzago, 1877)	*R. massiliae*	-	PP552645
504	Not available	*R. monacensis*	-	PP552646

Table S5. GenBank sequences generated from 120 strains, included in the phylogenetic analyses conducted in this study. *= Type strain. Genetic group abbreviations based on previous studies [7, 37]: CG = Canadensis Group; SFGI = Spotted Fever Group I; SFGII = Spotted Fever Group II (also known as Transitional Group, TRG); TG = Typhus Group; TIG = Tamurae/Ixodes Group.

**Taxa**	**Strain**	**Genetic group**	**GenBank accession**	
			ompA	***gltA***
'*Candidatus* Rickettsia andeanae'	T163	SFGI	-	GU169051
'*Candidatus* Rickettsia barbariae'	BL-2nd	SFGI	MW321563	MW321556
'*Candidatus* Rickettsia barbariae'	PoRbP88	SFGI	OR539312	OR539322
'*Candidatus* Rickettsia barbariae'	Sardinia	SFGI	EU272186	EU272185
'*Candidatus* Rickettsia goldwasserii'	B4	SFGI	HM136924	HM136923
'*Candidatus* Rickettsia hongyuanensis'	tick61	SFGI	OL335948	OK625738
'*Candidatus* Rickettsia jingxinensis'	F18	SFGI	MN550905	MN550898
'*Candidatus* Rickettsia jingxinensis'	Honghe-10	SFGI	OL856106	OL856123
'*Candidatus* Rickettsia jingxinensis'	Meixian-Hl-107	SFGI	MH932061	MH932016
'*Candidatus* Rickettsia laoensis'	447	SFGI	KT753293	KT753290
'*Candidatus* Rickettsia principis'	Weichang	SFGI	OP382385	OP382379
'*Candidatus* Rickettsia rioja'	DER204	SFGI	GQ404429	GQ404430
'*Candidatus* Rickettsia shennongii'	Honghe-3	SFGI	OL856102	OL856115
'*Candidatus* Rickettsia tarasevichiae'	A62p-20	CG	OM032872	OM032875
'*Candidatus* Rickettsia wissemanii'	G1329	SFGI	LT558853	LT558852
'*Candidatus* Rickettsia antechini'	-	SFGI	DQ372955	DQ372954
Rickettsia aeschlimannii	Baiyin-Ha14	SFGI	MH932058	MH932013
Rickettsia aeschlimannii	MC16*	SFGI	-	U59722
Rickettsia aeschlimannii	N381	SFGI	LC565690	LC565699
Rickettsia aeschlimannii	ZBSC1177	SFGI	MZ420684	MZ420699
Rickettsia africae	ESF5*	SFGI	CP001612	CP001612
Rickettsia akari	MK-Kaplan*	SFGII/TRG	-	U59717
Rickettsia amblyommatis	7_1	SFGI	MW741995	MW741984
Rickettsia amblyommatis	An13*	SFGI	DQ517292	CP015012
Rickettsia argasii	T170*	SFGI	JQ727681	LAOQ01000003
Rickettsia asembonensis	F82	SFGII/TRG	-	JN315974
Rickettsia asembonensis	LER197	SFGII/TRG	-	MK923733
Rickettsia asembonensis	NMRCii*	SFGII/TRG	-	JWSW01000078
Rickettsia asiatica	IO-1*	Uncertain	-	AF394901
Rickettsia australis	Cutlack	SFGII/TRG	CP003338	CP003338
Rickettsia australis	Philips*	SFGII/TRG	-	U59718
Rickettsia australis	PHS	SFGII/TRG	AF149108	
Rickettsia canadensis	2678*	CG	EF160122	U59713
Rickettsia canadensis	CA410	CG	CP003304	CP003304
Rickettsia canadensis	McKiel	CG	CP000409	
Rickettsia conorii subsp. *caspia*	A167*	SFGI	U43791	ARU59728
Rickettsia conorii subsp. *conorii*	Malish 7*	SFGI	NC003103	AE006914
Rickettsia conorii subsp. *heilongjiangensis*	HLJ-054*	SFGI	-	AF178034
Rickettsia conorii subsp. *heilongjiangensis*	Huaian-HFL	SFGI	ON600649	ON600643
Rickettsia conorii subsp. *israelensis*	ISTT CDC1*	SFGI	U43797	ITU59727
Rickettsia conorii subsp. *raoultii*	F226	SFGI	MF511262	MF511252
Rickettsia conorii subsp. *raoultii*	Khabarovsk*	SFGI	AH015610	DQ365804
Rickettsia conorii subsp. *raoultii*	LYG175	SFGI	JQ792142	JQ792115
Rickettsia conorii subsp. *raoultii*	LYG244	SFGI	JQ792146	JQ792119
Rickettsia conorii subsp. *raoultii*	LYG51	SFGI	JQ792138	JQ792111
Rickettsia conorii subsp. *raoultii*	LYG575	SFGI	JQ792153	JQ792124
Rickettsia conorii subsp. *raoultii*	LYG624	SFGI	JQ792155	JQ792125
Rickettsia conorii subsp. *raoultii*	M198	SFGI	MF511260	MF511250
Rickettsia conorii subsp. *raoultii*	M30	SFGI	MF511254	MF511244
Rickettsia conorii subsp. *raoultii*	N12	SFGI	MN550900	MN550894
Rickettsia conorii subsp. *raoultii*	N21	SFGI	MN550901	MN550895
Rickettsia conorii subsp. *raoultii*	N250	SFGI	MF511263	MF511253

Table S5. (Continued).

Rickettsia conorii subsp. *raoultii*	N34	SFGI	MN550902	MN550896
Rickettsia conorii subsp. *raoultii*	NH3-t189	SFGI	MW430420	MW430417
Rickettsia conorii subsp. *raoultii*	T23	SFGI	JQ798904	MW422251
Rickettsia conorii subsp. *raoultii*	WYG24	SFGI	JQ792158	JQ792128
Rickettsia fournieri	AUS118*	SFGI	KF666477	KF666471
Rickettsia gravesii	BWI1*	SFGI	DQ269437	DQ269435
Rickettsia helvetica	C9P9*	Uncertain	-	U59723
Rickettsia helvetica	Om74	Uncertain	-	OQ866615
Rickettsia helvetica	Om75	Uncertain	-	OQ092468
Rickettsia helvetica	Put117	Uncertain	-	OQ866616
Rickettsia helvetica	Skh7	Uncertain	-	OQ209950
Rickettsia honei subsp. *honei*	RB*	SFGI	AF018075	AF022817
Rickettsia honei subsp. *honei*	TT-118	SFGI	-	TTU59726
Rickettsia honei subsp. *marmionii*	KB*	SFGI	DQ309096	AY737684
Rickettsia hoogstraalii	Croatica*	SFGII/TRG	-	FJ767737
Rickettsia hulinii	HL-93*	SFGI	-	AF172943
Rickettsia japonica	PMK	SFGI	DQ909072	DQ909073
Rickettsia japonica	YH*	SFGI	D28766	AP011533
Rickettsia lusitaniae	CEVDIPoTiRo*	SFGII/TRG	JQ771935	JQ771933
Rickettsia massiliae	BjRt107	SFGI	MW026207	MW026225
Rickettsia massiliae	BjRt146	SFGI	MW026198	MW026222
Rickettsia massiliae	BzRt57	SFGI	MW026203	MW026237
Rickettsia massiliae	Mtu1*	SFGI	U43799	U59719
Rickettsia monacensis	H166	TIG	OL687187	OL687218
Rickettsia monacensis	H188	TIG	OL687182	OL687213
Rickettsia monacensis	H56	TIG	OL687177	OL687207
Rickettsia monacensis	IrR-Munich*	TIG	AH015165	CBUA010000018
Rickettsia monacensis	PoHuR34655	TIG	OK504619	OK504620
Rickettsia montanensis	VR-611*	SFGI	U43801	
Rickettsia monteiroi	Intervales*	CG	MK166031	FJ269035
Rickettsia parkeri	Maculatum20*	SFGI	U43802	U59732
Rickettsia peacockii	Skalkaho*	SFGI	AH013412	AY590152
Rickettsia prowazekii	Breinl*	TG	-	U20244
Rickettsia prowazekii	NMCR-Madrid	TG	-	CP004888
Rickettsia rhipicephali	376*	SFGI	U43803	U59721
Rickettsia rickettsii	Iowa*	SFGI	CP018914	CP000766
Rickettsia sibirica subsp. *mongolitimonae*	HA-91*	SFGI	U43796	U59731
Rickettsia sibirica subsp. *mongolitimonae*	Hyma226	SFGI	OQ123675	OQ123694
Rickettsia sibirica subsp. *sibirica*	246*	SFGI	U43807	U59734
Rickettsia slovaca	13B*	SFGI	U43808	CP002428
Rickettsia slovaca	AK	SFGI	MW922579	MW922559
Rickettsia slovaca	JK	SFGI	MW922580	MW922552
Rickettsia slovaca	NH	SFGI	MW922584	MW922556
Rickettsia sp.	18mN	-	MW741998	MW741994
Rickettsia sp.	CDC-PSFJC880	-	MN548859	MN581988
Rickettsia sp.	CDC-RconJC480	-	MN548866	MN581991
Rickettsia sp.	CT36	-	LC565679	LC565696
Rickettsia sp.	D025	-	AY543681	AY548828
Rickettsia sp.	JordanAC128-R	-	AB795210	AB795201
Rickettsia sp.	KS-Ck3	-	MT482549	MT482546
Rickettsia sp.	Madagascar	-	AB795206	AB795183
Rickettsia sp.	MAR2019-MRH	-	MZ015009	MT793831
Rickettsia sp.	ZambiaAS124-R	-	AB795209	AB795200
Rickettsia sp.	ZambiaAS62-R	-	AB795205	AB795175
Rickettsia tamurae subsp. *buchneri*	ISO7*	TIG	JFKF01000169	JFKF01000076
Rickettsia tamurae subsp. *tamurae*	1994-ISE6	TIG	LC388793	LC388786
Rickettsia tamurae subsp. *tamurae*	AT-1*	TIG	DQ103259	AF394896

Table S5. (Continued).

Rickettsia tamurae subsp. *tamurae*	Ate-1347	TIG	LC379464	LC379435
Rickettsia tillamookensis	Tillamook23*	SFGII/TRG	-	CP060138
Rickettsia typhi	CFDVUAM001	TG	-	MN544248
Rickettsia typhi	Wilmington*	TG	-	U59714
Rickettsia vini	Boshoek1	SFGI	MT062907	MT062909
Rickettsia vini	Breclav*	SFGI	KT326194	KT187394
Uncultured *Rickettsia* sp.	MA331AMAb	-	LC775598	LC775874
Uncultured *Rickettsia* sp.	MA341AFN	-	LC775594	LC775877
Uncultured *Rickettsia* sp.	QH-161	-	MG598413	MG598409
Uncultured *Rickettsia* sp.	QH-52	-	MG598411	MG598407
Uncultured *Rickettsia* sp.	tick26	-	OL335946	OK625736

Table S6. Best partition scheme and substitution model(s) for both datasets used in phylogenetic analyses, chosen according to BIC. The number of sequences included in each alignment appears in the second column, with those newly obtained for this study indicated between brackets. Ordinal numbers in superscript represent the three-codon positions for both protein-coding genes.

Gene dataset	Number of sequences	Predefined partitions	Best partition scheme	Best substitution model
ompA	409 (311)	3 partitions: *ompA*^{1st}<br>*ompA*^{2nd}<br>*ompA*^{3rd}	2 partitions: *ompA*^{1st} plus ^{2nd}<br>*ompA*^{3rd}	TVM+F+G4<br>K3Pu+F+G4
gltA	387 (270)	3 partitions: *gltA*^{1st}<br>*gltA*^{2nd}<br>*gltA*^{3rd}	3 partitions: *gltA*^{1st}<br>*gltA*^{2nd}<br>*gltA*^{3rd}	TIM3+F+G4<br>K2P+G4<br>K3Pu+F

Table S7. Comparison of the prevalence of three *Rickettsia* species in different periods in Castilla y León. The period 2018–2022 corresponds to this study and 1997–2002 corresponds to a previous study [14].

Species/period	2018–2022	1997–2002
Rickettsia slovaca	83/498 (16.67%)	42/193 (21.76%)
Rickettsia aeschlimanii	79/498 (15.86%)	35/193 (18.13%)
Rickettsia conorii subsp. *raoultii*	69/498 (13.86%)	23/193 (11.92%)
